# Clear aligner interface properties, oral microbial ecology, and strategies for biofilm control and caries-risk mitigation: a scoping review

**DOI:** 10.3389/fcimb.2026.1951083

**Published:** 2026-10-23

**Authors:** Zhuoyue Li, Yuanchao Yang, Bingshi Liu, Jiaman Sun, Xinchen Tang, Yan Yang

**Affiliations:** 1 Xiangya Stomatological Hospital and Xiangya School of Stomatology, Central South University, Changsha, China; 2 Hunan Key Laboratory of Oral Health Research, Changsha, China

**Keywords:** antibiofilm strategies, caries prevention, clear aligners, interface properties, oral microbial ecology

## Abstract

**Background:**

Clear aligners create an aligner–tooth interface that may retain saliva, microorganisms, and dietary substrates. Evidence linking interface properties, oral microecological responses, and preventive interventions remains fragmented.

**Objective:**

To map and synthesize evidence on clear aligner interface properties and microbial adhesion, oral microbial ecology during clear aligner wear, and strategies for biofilm control and caries-risk mitigation.

**Methods:**

Following Joanna Briggs Institute methodology and PRISMA-ScR guidance, PubMed, Embase, and Web of Science Core Collection were searched from inception to August 31, 2026, without language restrictions. Backward and forward citation searching supplemented database searches. Original clinical, *in vitro*, and ex vivo studies involving active clear aligner treatment, associated materials or interfaces, or clear-aligner-specific interventions were eligible when they reported microbial, biofilm, microbiome, metabolomic, caries-related, or mineral outcomes. Two reviewers independently screened records; two authors charted and verified data. Findings were synthesized descriptively within three evidence domains.

**Results:**

Of 988 records, 132 reports were included. Because reports could contribute to more than one domain, 62 informed interface properties and microbial adhesion, 47 oral microbial ecology during wear, and 76 biofilm control and caries-risk mitigation. No single polymer, manufacturing method, or surface parameter consistently predicted microbial adhesion or biofilm formation. Outcomes reflected interactions among material characteristics, local topography, salivary conditioning, aging and exposure, microbial species, and experimental models. Clinical microbial responses were compartment- and time-dependent. Salivary and tooth-associated communities often remained relatively stable at the community level, whereas the inner aligner surface sometimes exhibited localized taxonomic, functional, metabolic, and pH-related changes. Investigated interventions included cleaning, antimicrobial and antifouling interface modifications, anticaries and remineralizing approaches, probiotics, and environment-responsive systems. Although many strategies reduced microbial burden, biofilm-related outcomes, acidogenicity, or mineral loss under tested conditions, evidence was predominantly short-term and preclinical. Assessments of durability, material performance, and safety were heterogeneous.

**Conclusions:**

Clear aligner–microbiota interactions appear context-dependent rather than uniformly favorable or unfavorable. Evidence neither establishes causal relationships between interface or microecological changes and clinical disease nor identifies a universally superior intervention. More standardized, clinically representative evidence may clarify their clinical relevance and inform preventive management during clear aligner treatment.

## 1 Introduction

Clear aligners have emerged as a rapidly developing modality in contemporary orthodontic treatment. Compared with conventional fixed appliances, clear aligners offer favorable esthetics, greater comfort, removability, and easier cleaning, and are therefore increasingly used in both adolescent and adult orthodontic patients (Martínez-Hernández et al., 2026). Several clinical studies and systematic reviews have suggested that patients treated with clear aligners generally show lower plaque indices, milder gingival inflammation, and better oral hygiene maintenance, and that their overall disturbance to the oral microbial ecology may be less pronounced than that associated with fixed appliances (Alhuwaizi et al., 2026; Tabone et al., 2026).

However, the removability of clear aligners does not mean that they are free from microecological risks. During treatment, patients usually need to wear aligners for prolonged periods each day. Their inner surfaces continuously cover tooth surfaces and remain in close contact with saliva, gingival crevicular fluid, the acquired pellicle, food residues, and oral microorganisms (de Souza Schacher et al., 2025). This interfacial environment may be relatively enclosed and saliva-retentive during wear, which may favor microbial retention and biofilm development (Yan et al., 2021; Rusu et al., 2026).

The core issue in clear aligner-associated microecology lies not only in whether the appliance increases plaque accumulation, but also in whether it alters the local interfacial environment (Tabone et al., 2026). The clear aligner surface is not an inert material plane, but a dynamic ecological interface shaped by material properties, manufacturing procedures, intraoral aging, salivary pellicle formation, cleaning behaviors, and patient habits (Gracco et al., 2009; Chen et al., 2025). Surface roughness, wettability, surface free energy, and chemical functional groups may influence salivary protein adsorption and initial bacterial colonization (Quirynen and Bollen, 1995; Bollen et al., 1997; Martínez Gil-Ortega et al., 2025). As wear time increases, microcracks, abrasion, pigment deposition, and chemical aging may develop on the aligner surface, thereby further modifying microbial adhesion behavior (Schuster et al., 2004; Gracco et al., 2009; Lira et al., 2023; Eslami et al., 2024). In addition, clear aligners are often used in combination with composite resin attachments (Jiang et al., 2023). The margins of attachments, gingival margins, and interproximal areas are prone to plaque retention and may increase the risk of white spot lesions, dental caries, and gingivitis when oral hygiene is poorly controlled (Alqarni et al., 2025).

In recent years, advances in 16S ribosomal RNA (16S rRNA) gene sequencing, metagenomics, metabolomics, and biomaterial characterization have shifted clear aligner-related research from the simple observation of plaque indices and periodontal clinical parameters toward an integrated assessment of material interfaces, biofilm architecture, microbial community composition, metabolic function, and host responses (Peregrina et al., 2025). hemic At the same time, interventions targeting biofilms on clear aligner surfaces have expanded beyond conventional brushing and chemical cleaning (Hu et al., 2026). Investigated approaches include antimicrobial coatings, anti-adhesive interfaces, remineralizing materials, pH-responsive systems, and probiotics (Xie et al., 2020; Sun et al., 2022; Gong et al., 2023; Yan et al., 2023; Wu et al., 2024). liter Recent reviews have separately examined oral hygiene and periodontal outcomes, white spot lesions and caries-associated bacteria, home biofilm management, and oral microbiome changes (Raghavan et al., 2023; Pardo et al., 2024; Alhuwaizi et al., 2026; Tabone et al., 2026). Other reviews have focused on marginal-edge design or broader relationships among aligner materials, oral health, and environmental considerations (Martínez-Hernández et al., 2026; Rusu et al., 2026). However, evidence concerning interface properties and microbial adhesion, microbial and metabolic changes during active wear, and biofilm-control and caries-risk-mitigation strategies remains distributed across separate bodies of literature. Mapping these complementary evidence streams within one framework may clarify how each domain has been studied and where connections among them remain insufficiently examined. Given the breadth and methodological heterogeneity of this evidence, a scoping review was selected to map the available literature and identify evidence gaps rather than to estimate a single pooled effect (Munn et al., 2018). Accordingly, this scoping review aimed to systematically map and synthesize evidence on (1) clear aligner interface properties and microbial adhesion, (2) changes in oral microbial ecology during clear aligner wear, and (3) strategies for biofilm control and caries-risk mitigation.

## 2 Methods

### 2.1 Review design

This scoping review was conducted in accordance with the Joanna Briggs Institute (JBI) methodology for scoping reviews and reported according to the Preferred Reporting Items for Systematic Reviews and Meta-Analyses Extension for Scoping Reviews (PRISMA-ScR) (Tricco et al., 2018; Peters et al., 2020; Sarkis-Onofre et al., 2021). The completed PRISMA-ScR checklist is provided in **Supplementary Table S1**.

The review question and eligibility criteria were structured using the Population–Concept–Context (PCC) framework. The population component encompassed human participants receiving clear aligner treatment and experimental models involving clear aligner materials or interfaces. The concepts included material and interface characteristics, microbial adhesion and biofilm formation, oral microbial and metabolic changes, and strategies for biofilm control or caries-risk mitigation. The context comprised relevant clinical and laboratory settings. This review was not prospectively registered.

### 2.2 Information sources and search strategy

A systematic literature search was conducted in PubMed, Embase via Embase.com, and Web of Science Core Collection from database inception up to August 31, 2026, with no language restrictions. Search terms related to clear aligners were combined with terms related to microbial adhesion, biofilms, oral microbiota or microbiome, metabolomics, dental caries, white spot lesions, and relevant preventive or therapeutic approaches. Controlled vocabulary and free-text terms were adapted to the syntax of each database. The complete database-specific search strategies, search dates, and numbers of records retrieved are provided in **Supplementary Table S2**. Search methods were reported in accordance with PRISMA-S (Rethlefsen et al., 2021).

Supplementary searching comprised backward citation searching of eligible reports, reference-list checking of relevant reviews, and forward citation searching of eligible reports using the “Cited by” function in Google Scholar (Hirt et al., 2024). Records identified through supplementary searching were deduplicated against the database results and assessed using the same eligibility criteria. Supplementary searches were last conducted on August 31, 2026.

All retrieved records were imported into Zotero for reference management. Automated duplicate detection was followed by manual verification before screening.

### 2.3 Eligibility criteria

Original clinical, *in vitro*, or ex vivo studies were eligible if they investigated clear aligners or clear overlay appliances explicitly used for active orthodontic tooth movement; associated trays, materials, interfaces, or attachments; or a clear-aligner-specific intervention, and met at least one of the following conditions: (1) reported a direct microbial, biofilm, microbiota, microbiome, or metabolomic outcome; or (2) evaluated a clear-aligner-specific cleaning, disinfection, antimicrobial, antibiofilm, anti-adhesive, probiotic, caries-preventive, or remineralizing intervention using a microbial, biofilm, caries-related, or mineral outcome.

Material or interface studies were required to evaluate a defined material or interface factor in relation to a direct microbial or biofilm endpoint. Clear-aligner-specific anticaries or remineralizing interventions were eligible when they reported a mineral or caries-related outcome, even without a direct microbial endpoint. Studies comparing fixed or other removable orthodontic appliances were eligible only when clear-aligner-specific data were separately extractable.

Studies reporting only white spot lesions, demineralization, decayed, missing, and filled teeth (DMFT), caries incidence, salivary pH or flow, plaque indices, or general periodontal indices were excluded unless they evaluated an eligible clear-aligner-specific preventive or remineralizing intervention. Studies of passive retainers or other removable appliances without an explicit active clear-aligner or active clear-overlay component were excluded. Studies focused solely on biomechanics, treatment accuracy, aesthetics, pain, comfort, or unrelated material characterization were also excluded.

Reviews, systematic reviews, meta-analyses, protocols without results, case reports or case series, editorials, letters, commentaries, and conference abstracts without sufficient methodological and outcome data were excluded from the primary evidence set. Reviews were used only to identify potentially eligible primary studies through supplementary searching. Animal-only reports were excluded; where an otherwise eligible report also contained an animal experiment, that component was retained only as ancillary preclinical evidence.

### 2.4 Study selection

Following duplicate removal, titles and abstracts were independently screened by two authors. The same authors independently assessed potentially eligible full-text reports. Disagreements were resolved through discussion until consensus was reached.

### 2.5 Data charting and evidence synthesis

Three structured, domain-specific data-charting tables were developed according to the three predefined evidence domains of the review. Rather than applying a single uniform set of variables to all included studies, each table contained data items relevant to the corresponding review question and study characteristics. Study identifiers were recorded using the first author and year of publication. Two authors independently charted data from the included reports, and uncertainties or discrepancies were resolved through discussion. Information that was applicable but not reported was recorded as “NR,” whereas items that were not applicable to a particular study were recorded as “N/A” (Pollock et al., 2023).

For studies addressing Clear Aligner Interface Properties and Microbial Adhesion, the charted data comprised study design or model, material or interface factor, interface characterization, microbial model, experimental conditions, adhesion or biofilm assessment methods, main outcomes, and key findings concerning the relationship between interface characteristics and microbial adhesion or biofilm formation.

For studies addressing Changes in Oral Microbial Ecology During Clear Aligner Wear, the charted data comprised study design, population and comparator, aligner system or exposure, sampling compartment, sampling time points and follow-up duration, analytical methods, microbial outcomes, and key findings regarding changes in oral microbial ecology.

For studies addressing Biofilm Control and Caries-Risk Mitigation, the charted data comprised study design or model, intervention category, intervention or strategy, proposed mechanism, microbial or experimental model, treatment or exposure conditions, primary outcomes, key efficacy findings, and reported evidence regarding durability, material performance, or safety.

Studies addressing more than one evidence domain were included in each relevant data-charting table, with information extracted according to the specific question addressed in that domain. Such cross-listing did not alter the number of unique included studies.

The charted data were tabulated and summarized descriptively within the three predefined evidence domains. No quantitative meta-analysis was undertaken.

## 3 Results

### 3.1 Search results, study selection, and evidence-domain distribution

Database searches identified 981 records, including 341 from PubMed, 307 from Embase, and 333 from Web of Science Core Collection. Supplementary backward and forward citation searching identified seven additional records, yielding 988 records in total. After removal of 490 duplicates, 498 unique records underwent title and abstract screening, of which 321 were excluded. Full-text retrieval was sought for 177 reports; two reports could not be retrieved, leaving 175 reports for eligibility assessment. Of these, 43 were excluded, including 36 because of an ineligible concept or outcome and seven because of an ineligible publication or report status. A total of 132 reports were included in the scoping review (**Figure 1**).

**Figure 1:**
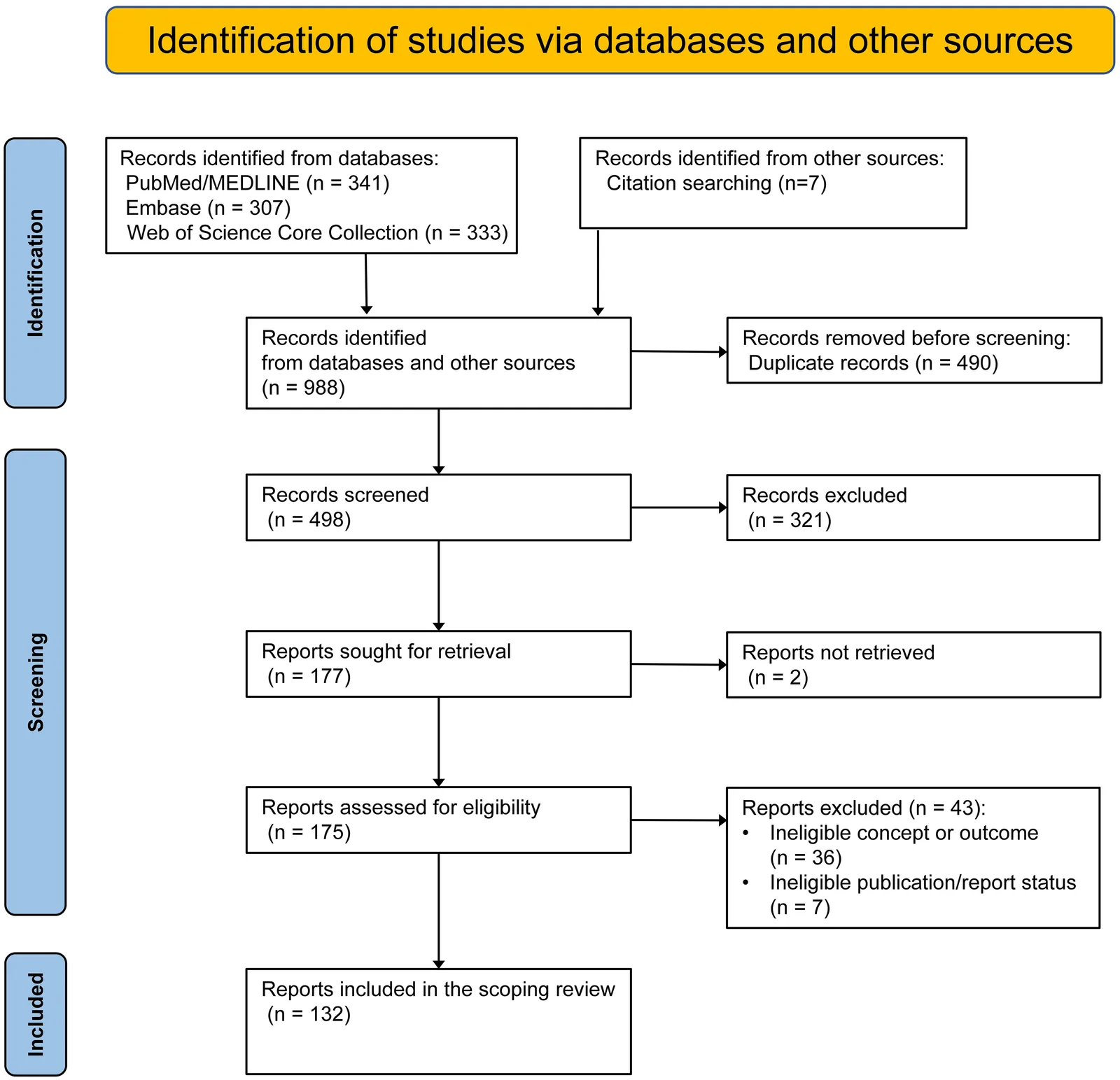
PRISMA-ScR flow diagram of report selection. Database searches identified 981 records, and citation searching identified seven additional records. After 490 duplicates were removed, 498 records were screened and 321 were excluded. Of the 177 reports sought for retrieval, two could not be retrieved. The remaining 175 reports were assessed for eligibility; 43 were excluded because of an ineligible concept or outcome (n = 36) or an ineligible publication or report status (n = 7). Overall, 132 reports were included in the scoping review.

The included reports were classified into three predefined evidence domains: Clear Aligner Interface Properties and Microbial Adhesion, Changes in Oral Microbial Ecology During Clear Aligner Wear, and Biofilm Control and Caries-Risk Mitigation. Because individual reports could address more than one domain, the domain-specific counts exceeded the number of unique included reports. The total of 62 reports contributed to the interface domain, 47 reports contributed to the oral microbial ecology domain, and 76 reports contributed to the biofilm-control and caries-risk-mitigation domain (**Figure 2**).

**Figure 2:**
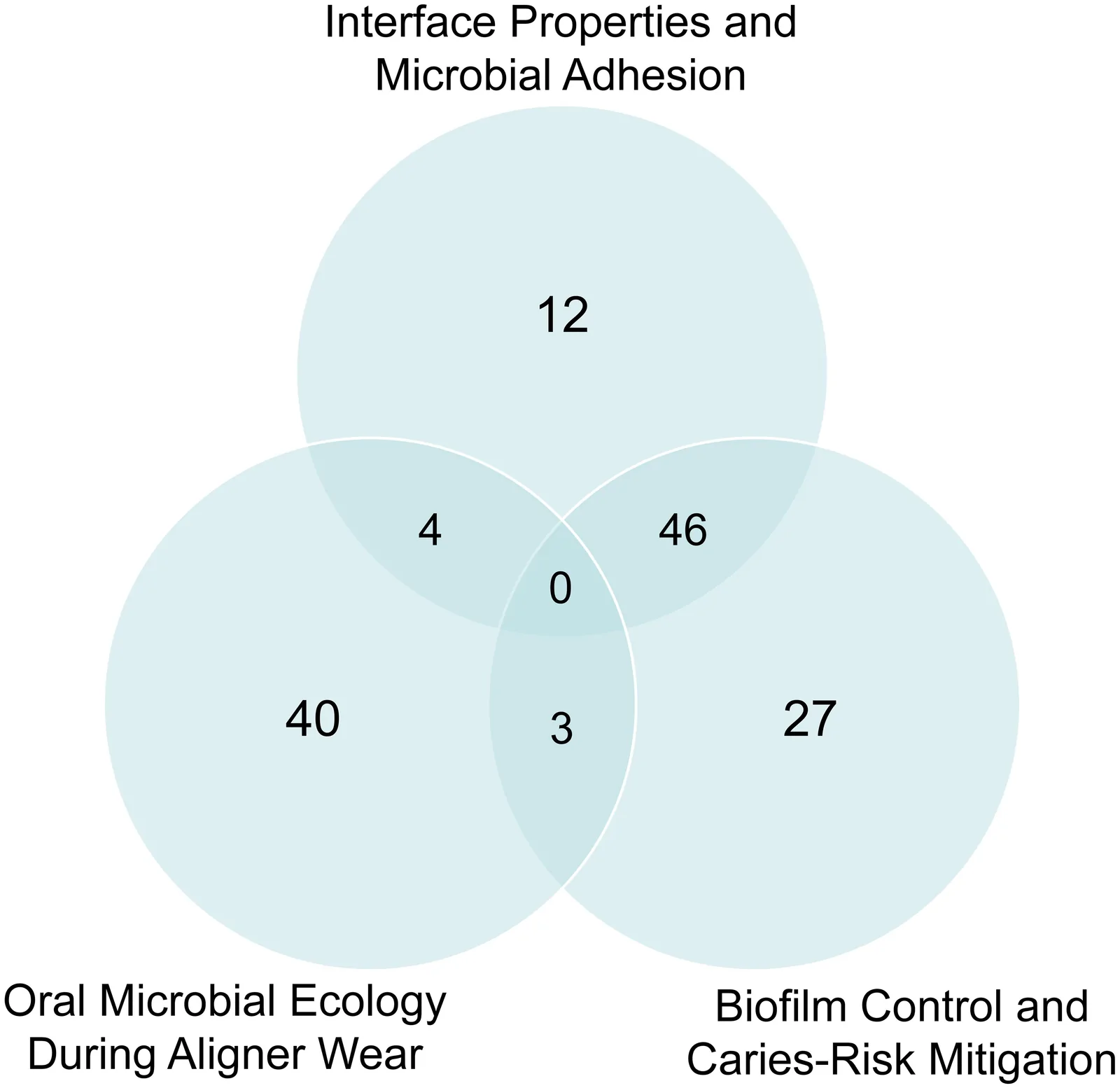
Distribution of included reports across the three thematic domains. The Venn diagram shows the classification of 132 reports into interface properties and microbial adhesion, oral microbial ecology during aligner wear, and biofilm control and caries-risk mitigation. Numbers indicate mutually exclusive regions: 12, 40, and 27 reports addressed only the respective individual domains; four addressed both interface properties and oral microbial ecology, 46 addressed both interface properties and biofilm control and caries-risk mitigation, and three addressed both oral microbial ecology and biofilm control and caries-risk mitigation. No report covered all three domains.

**Table 1** provides a comparative overview of the study designs, aligner or material characteristics, microbiological and analytical methods, outcomes, and principal evidence patterns across the three domains. Detailed study-level data are provided in **Supplementary Tables S3–S5**.

**Table 1:** Comparative overview of the evidence mapped across the three review domains.

Evidence domain	Predominant study designs and settings	Main aligner, material, or exposure characteristics	Microbiological and analytical methods	Main outcomes	Overall evidence pattern and principal gaps
Clear aligner interface properties and microbial adhesion (n = 62 reports)	Mainly *in vitro* material–microbial studies. Fourteen reports primarily evaluated interface characteristics; the remainder provided complementary interface evidence, mainly within intervention studies. *In situ* and clinical intraoral-wear evidence was limited.	Thermoformed and directly printed materials; polymer composition; surface roughness and morphology; wettability and surface free energy; surface charge and chemistry; salivary conditioning; aging, wear, and cleaning-related changes.	Profilometry, AFM, SEM, contact-angle analysis, and spectroscopy, combined with monospecies, defined polymicrobial, or saliva-derived microbial models under static or dynamic exposure conditions.	Interface characteristics; microbial adhesion and surface distribution; CFU; microbial viability and metabolic activity; biofilm biomass, morphology, and architecture.	No single polymer, manufacturing method, or surface parameter consistently predicted microbial colonization. Comparability was limited by heterogeneous materials, conditioning and aging protocols, microbial models, and assessment methods.
Oral microbial ecology during clear aligner wear (n = 47 reports)	Thirty-seven reports used longitudinal, repeated-measures, before-and-after, or crossover designs; 10 used cross-sectional or single-time-point sampling. Studies sampled one or multiple oral compartments.	Clear aligner exposure ranged from 24 h to 1.5 years, with some post-treatment follow-up. Sampling involved saliva, aligner-associated biofilm or fluid, supragingival plaque, subgingival plaque, and mucosal sites.	Culture and CFU enumeration; targeted qPCR or DNA arrays; 16S rRNA gene sequencing; shotgun metagenomics; microscopy; biochemical measurements; targeted or untargeted metabolomics.	Microbial load; alpha and beta diversity; community composition; selected taxa; predicted or gene-level functional potential; metabolites; local pH.	Responses were compartment- and time-dependent. Salivary and tooth-associated communities were often relatively stable, whereas aligner surfaces showed more localized changes. Comparability was limited by variation in sampling sites, time points, populations, and analytical methods.
Biofilm control and caries-risk mitigation (n = 76 reports)	Forty-six *in vitro* material–microbial reports; 7 reports incorporating ex vivo enamel models; 9 with ancillary animal or preliminary human components; 13 clinical interventions; and 1 retrieved-aligner rechallenge study.	Conventional cleaning and decontamination; antimicrobial interfaces; anti-adhesive or antifouling modifications; anticaries or remineralizing systems; probiotics; environment-responsive strategies.	Monospecies, defined multispecies, and saliva- or plaque-derived biofilms; material testing; pH cycling; ex vivo enamel models; selected clinical microbial, biofilm, and oral-health assessments.	Microbial burden, adhesion, viability, biomass, metabolic activity, and acidogenicity; mineral loss or gain; durability; optical and mechanical performance; safety and cytocompatibility.	Many strategies improved microbial, biofilm, or mineral outcomes under tested conditions, but evidence was mainly short-term and preclinical. Heterogeneous models and endpoints precluded direct ranking and definitive conclusions about long-term clinical benefit.

Reports could contribute to more than one evidence domain; therefore, domain-specific counts are not mutually exclusive. AFM, atomic force microscopy; CFU, colony-forming unit; qPCR, quantitative polymerase chain reaction; SEM, scanning electron microscopy.

### 3.2 Interface properties of clear aligners and microbial adhesion

During wear, the clear aligner interface is in direct contact with teeth, saliva, and oral microbial communities, making it a critical surface that influences initial microbial adhesion and biofilm formation (Yan et al., 2021). This surface is determined not only by the physicochemical properties of the material itself, but also by multiple factors related to clinical processing, aging, and use (Gao et al., 2025; Goracci et al., 2025). Among the 62 reports classified within this domain, 14 primarily investigated interface characteristics in relation to microbial outcomes, whereas the remaining studies provided complementary interface evidence, mainly in studies of biofilm-control strategies. Overall, microbial adhesion was not consistently determined by a single polymer, manufacturing method, or surface parameter, but reflected the combined effects of material properties, local topography, salivary conditioning, exposure history, microbial species, and experimental model. The relevant studies are summarized in **Supplementary Table S3**.

#### 3.2.1 Material composition and manufacturing procedures

The interface properties of clear aligners are first determined by their material composition and manufacturing procedures. Most clinically used clear aligners are fabricated from thermoplastic polymers, including glycol-modified polyethylene terephthalate (PETG), thermoplastic polyurethane (TPU), polyurethane-based materials, and multilayer composite sheets (Gao et al., 2025). With the development of digital orthodontics and additive manufacturing, direct three-dimensional (3D) printing of clear aligners has become an emerging research focus (Chen et al., 2025). Different materials vary in elastic modulus, water sorption, transparency, thermal stability, surface chemical composition, and aging behavior (Lira et al., 2023; Eslami et al., 2024). These differences may influence microbial adhesion and biofilm formation (Goracci et al., 2025).

Thermoforming is currently the main method used to fabricate clear aligners (Martínez-Hernández et al., 2026). During this process, a heated sheet is pressed to adapt to the dental cast, which may result in uneven material thickness, localized stretching, and heterogeneous surface relief (Lira et al., 2023). Rodríguez Fernández et al. observed preferential colonization of irregular thermoformed regions by *Streptococcus oralis* and *Actinomyces viscosus* (Rodríguez Fernández et al., 2024). By contrast, 3D-printed aligners offer additional interface variables, including their layer lines, post-curing degree, residual resin, and surface finishing (Wu et al., 2024). Chen et al. compared directly 3D-printed and thermoformed aligners and found differences among materials in roughness, optical properties, color stability, and *Streptococcus mutans* adhesion (Chen et al., 2025). However, direct printing did not consistently reduce microbial adhesion compared with thermoforming. After artificial-saliva aging, the roughest printed material did not show the greatest bacterial adhesion, and one directly printed resin showed broadly comparable adhesion to conventional thermoformed materials after 7 days. Tektas et al. likewise found no significant differences in initial adhesion or mature saliva-derived microcosm biofilms among four thermoplastic materials despite differences in surface characteristics (Tektas et al., 2020). Gao et al. further compared short-term microbial dynamics on PETG and TPU materials and suggested that material selection may influence salivary pH changes as well as microbial fluctuations on aligner surfaces and in supragingival plaque (Gao et al., 2025). However, because interface properties were not simultaneously characterized, these microbial differences could not be attributed to a single material feature.

Overall, broad categories such as PETG, TPU, thermoformed, or directly printed are insufficient alone to predict microbial colonization.

#### 3.2.2 Surface roughness and microscale morphology

Surface roughness and microscale morphology are important factors influencing microbial adhesion (Martínez Gil-Ortega et al., 2025). For clear aligners, surface depressions, scratches, cracks, micropores, and worn areas may all become initial sites for bacterial retention and biofilm maturation (Galisteu-Luiz et al., 2026). Similar to tooth surfaces and restorative materials, changes in the microscale morphology of clear aligner surfaces may reduce cleaning efficiency and promote persistent colonization by bacteria and fungi (Quirynen and Bollen, 1995; Bollen et al., 1997). Available evidence suggests that the geometry and distribution of local surface features may be more relevant than mean roughness alone. Low et al. showed that naturally formed multispecies biofilms preferentially accumulated in recessed and sheltered regions of aligner specimens, whereas flatter and more exposed regions retained less biofilm (Low et al., 2011). Similar preferential colonization has been reported at internal folds, surface reliefs, and finishing defects (Rodríguez Fernández et al., 2024; de Souza Schacher et al., 2025). In attachment-related models, rougher finishing surfaces also supported greater multispecies biofilm accumulation (Mota et al., 2026). Nevertheless, a higher numerical roughness value did not consistently correspond to greater adhesion. This association was absent in some comparative studies, including Tektas et al. and Chen et al (Tektas et al., 2020; Chen et al., 2025). Surface morphology should therefore be interpreted together with surface chemistry, microbial species, and exposure conditions.

Clear aligners are not static in the oral cavity. During wear, the material may be affected by salivary immersion, occlusal friction, insertion and removal stress, dietary pigments, acidic beverages, temperature changes, and cleaning procedures. In an *in vivo* study, Eslami et al. observed that both directly printed aligners and Invisalign aligners showed changes in surface roughness and porosity parameters after short-term intraoral use (Eslami et al., 2024). Lira et al. also reported that intraoral exposure can affect the chemical, physical, mechanical, and morphological properties of clear aligners (Lira et al., 2023).

Cleaning behavior may also participate in interfacial remodeling. Hu et al. compared multiple cleaning methods and found that brushing, especially when combined with chemical cleaning, effectively reduced *S. mutans* biofilms, whereas surface roughness increased in some brushing groups after repeated cleaning (Hu et al., 2026). In addition, tobacco exposure may alter the surface characteristics of aligners. Galisteu-Luiz et al. showed *in vitro* that cigarette smoke exposure increased the surface roughness of Invisalign aligners and enhanced bacterial adhesion (Galisteu-Luiz et al., 2026). These findings indicate that surface roughness is not a fixed material attribute of clear aligners but a dynamic outcome jointly determined by processing, wear, cleaning, and lifestyle-related behaviors.

#### 3.2.3 Wettability and surface free energy

Wettability and surface free energy influence salivary conditioning and initial bacterial adhesion (Martínez Gil-Ortega et al., 2025). After a clear aligner is placed in the oral cavity, its surface is rapidly covered by salivary proteins, glycoproteins, enzymes, and mineral ions, forming a pellicle-like interfacial layer (Quirynen and Bollen, 1995). In fact, bacteria do not always contact the bare material surface directly but may adhere indirectly through protein receptors, glycan structures, and ionic bridging within the acquired pellicle (de Souza Schacher et al., 2025). Therefore, material wettability, surface free energy, and protein adsorption may collectively influence the environment for early microbial colonization.

Different bacteria do not respond identically to hydrophilic and hydrophobic surfaces. Martínez Gil-Ortega et al. compared the wettability, surface free energy, and bacterial adhesion behavior of different clear aligner systems and found differences in contact angle, surface free energy, and metabolic activity of different strains among Spark, Invisalign, and Smile materials. Under dynamic-flow conditions, neither greater hydrophilicity nor greater hydrophobicity consistently reduced adhesion across all microorganisms (Martínez Gil-Ortega et al., 2025). In an artificial saliva environment, de Souza Schacher et al. observed that both *S. mutans* and *Candida albicans* adhered to the inner surface of clear aligners, with microbial accumulation also influenced by local surface morphology (de Souza Schacher et al., 2025).

From a clinical perspective, wettability and surface free energy are important not only for explaining bacterial adhesion mechanisms, but also for guiding subsequent anti-adhesive material design. For example, hydrophilic or zwitterionic interfaces can reduce protein adsorption by forming a stable hydration layer, thereby decreasing initial bacterial adhesion. Hydrophilic polysaccharide and zwitterionic modifications have therefore been explored as hydration-mediated antifouling strategies (Park et al., 2018; Wu et al., 2024; Paik et al., 2025).

#### 3.2.4 Surface charge and chemical functional groups

The surface charge state and chemical functional groups of materials also participate in bacterial adhesion. Bacterial surfaces are usually negatively charged. When positively charged functional groups are introduced onto a material interface, they may enhance contact through electrostatic interactions and may also disrupt bacterial cell membrane structures, thereby producing antimicrobial effects (Chen et al., 2026a).

Quaternary ammonium-containing materials, quaternized tannic acid coatings, N-halamine coatings, and some antimicrobial peptide coatings can achieve contact killing or sustained bacterial inhibition through cationic or reactive chemical groups (Nemec et al., 2025; Li et al., 2026b). In contrast, zwitterionic groups such as carboxybetaine methacrylate (CBMA) and 2-methacryloyloxyethyl phosphorylcholine (MPC) can reduce protein and microbial adhesion through strong hydration (Wu et al., 2024; Paik et al., 2025). More recently, pH-responsive and charge-switching systems have been designed to enhance antimicrobial activity under acidic biofilm conditions rather than provide continuous broad-spectrum activity (Sun et al., 2022; Li et al., 2026b). However, these modifications usually alter several interface properties simultaneously, making it difficult to attribute their effects to surface charge alone.

Overall, the available evidence supports a multifactorial interface model in which material composition and manufacturing establish the initial surface state, local microscale morphology provides retention niches, and salivary conditioning, surface chemistry, aging, and environmental exposure further modify microbial interactions. No single surface parameter consistently predicts clear-aligner biofilm formation.

### 3.3 Changes in oral microbial ecology during clear aligner wear

During clear aligner wear, the inner aligner surface and the narrow aligner–tooth space form additional ecological compartments in which salivary components, retained dietary substrates, and surface-associated microorganisms interact. Microbial responses within these compartments vary with the sampled oral site, duration of wear, oral hygiene, dietary exposure, and patient characteristics (Yan et al., 2021; Rouzi et al., 2023). Current evidence therefore indicates localized, site-specific, and time-dependent ecological changes rather than a uniform and sustained disruption of the entire oral microbiome (Tabone et al., 2026).

A total of 47 reports contributed evidence to this domain: 42 were primarily classified in the oral microbial ecology domain, whereas 5 contributed additional clinical microbial evidence from interface or intervention studies. Of these, 37 used longitudinal, repeated-measures, before-after, or crossover designs, and 10 relied on cross-sectional or single-time-point sampling. Sampling was heterogeneous and overlapping: saliva was analyzed in 16 reports, aligner-associated plaque, biofilm, fluid, or material surfaces in 15, supragingival plaque in 13, subgingival plaque in 15, and oral mucosal sites in 1 report; 18 reports sampled more than one compartment. Schedules ranged from repeated observations within a single 24 h wearing cycle to 1.5 years of active treatment and 1 year after treatment completion (Yan et al., 2021; Kredig et al., 2025), while study populations ranged from children aged 7 years and mixed-dentition cohorts to adults aged 64 years (Cenzato et al., 2022; Chen et al., 2024; Peregrina et al., 2025). Sequencing-based approaches were used in 23 reports, predominantly 16S rRNA gene sequencing, whereas the remaining studies used culture or colony counts, species-targeted quantitative polymerase chain reaction (qPCR) or DNA arrays, microscopy, biochemical measurements, or combinations of these methods. Functional assessment ranged from 16S-based pathway prediction and shotgun metagenomic gene profiling to 1 targeted and 3 untargeted metabolomic workflows, including paired microbiome-metabolome analyses (Wang et al., 2019, 2024; Song et al., 2023; Gong et al., 2025; Xie et al., 2025a, 2025b). Detailed methodological characteristics are provided in **Supplementary Table S4**.

#### 3.3.1 Microbial diversity and community structure

Saliva, supragingival plaque, subgingival plaque, mucosal plaque, and aligner-associated plaque represent biologically distinct microbial habitats and should not be interpreted interchangeably. Across longitudinal sequencing studies, alpha diversity showed no consistent upward or downward trajectory. It remained broadly stable in several studies of saliva, supragingival plaque, and subgingival plaque, including adult, adolescent, and mixed-dentition cohorts followed for 3–6 months (Zhao et al., 2020; Chen et al., 2024; Wang et al., 2024; Gong et al., 2025; Nemec et al., 2025; Peregrina et al., 2025). A 3-month subgingival study showed only a nonsignificant downward trend in alpha diversity despite a significant change in community structure (Guo et al., 2018).

Decreases in richness or alpha diversity were more often reported in aligner-associated or selected mucosal communities, but these findings were not uniform. Inner-aligner diversity declined during 24 h without cleaning, and tray-associated richness and diversity decreased over 1–3 months as *Streptococcus* became more dominant (Yan et al., 2021; Rouzi et al., 2023). By contrast, a 24-h PETG–TPU crossover study found no sustained alpha-diversity change across saliva, supragingival plaque, or aligner surfaces (Gao et al., 2025). At oral mucosal sites, alpha diversity of the buccal community decreased at 6 months but returned toward baseline by 12 months, with no parallel trajectory across the palatal and dorsal-tongue sites (Jiao et al., 2025).

Beta diversity and community composition were likewise compartment dependent. Subgingival community structure changed over 3 months in one single-arm cohort, whereas other longitudinal studies found little or no overall change in subgingival, supragingival, or salivary communities (Guo et al., 2018; Rouzi et al., 2023; Peregrina et al., 2025). Within multisite studies, variation among saliva, tooth-associated plaque, subgingival plaque, and tray plaque generally exceeded variation over time or between appliance groups (Shokeen et al., 2022; Peregrina et al., 2025). In contrast, aligner-tray communities were repeatedly distinguishable from tooth-associated plaque and changed during wear (Shokeen et al., 2022; Rouzi et al., 2023; Haug et al., 2024). Cross-sectional studies also detected appliance-group differences, but their single sampling points did not establish longitudinal treatment effects (Wang et al., 2019; Zheng et al., 2024).

Overall, the evidence did not identify a consistent whole-mouth diversity shift during clear aligner treatment. Relatively stable community-level indices in saliva and conventional dental plaque compartments coexisted with more dynamic, time-specific changes on the aligner surface and at selected mucosal sites.

#### 3.3.2 Changes in caries- and periodontal-associated microorganisms

Evidence on specific microorganisms came from analytically distinct sources. Culture and targeted qPCR studies estimated absolute or semiquantitative microbial loads, whereas 16S rRNA sequencing primarily described relative abundance within a community; these findings were therefore not treated as equivalent. In comparative culture or qPCR studies, salivary *Streptococcus mutans* and *Lactobacillus* levels were either similar between aligner and fixed-appliance groups during early treatment or less likely to reach high-caries-risk levels with aligners by 6 months (Sifakakis et al., 2018; Mummolo et al., 2020b, 2020a). A within-patient arch comparison likewise reported lower high-risk *S. mutans* and *Lactobacillus* levels in the aligner arch, although appliance type was inseparable from arch and sampling site (Giannini et al., 2025). In contrast, a randomized study in children found increased plaque *S. mutans* and decreased *Candida albicans* after clear aligner treatment. However, interpretation was limited by differences in treatment duration between the aligner and Z-spring groups (Kapicıi et al., 2025).

Sequencing studies showed that caries-associated changes were most apparent in selected compartments or risk subgroups rather than as a uniform community-wide pattern. During 24 h without cleaning, the inner aligner surface showed increased *Streptococcus* and Lactobacillales together with decreased *Actinomyces* and *Rothia*, and a separate longitudinal study found increasing *Streptococcus* dominance in tray plaque over 1–3 months (Yan et al., 2021; Rouzi et al., 2023). However, clear aligner users in mixed dentition showed no significant overall genus-level change and only a nonsignificant upward trend in *Lactobacillus* at 6 months, while another salivary cohort showed no substantial increase in caries-associated species (Chen et al., 2024; Nemec et al., 2025). In adolescents with poor plaque control, shotgun metagenomics detected increases in caries-associated species and biofilm-related functions together with rapid white spot lesion development, whereas the overall supragingival community remained broadly stable (Wang et al., 2024).

Periodontal-associated findings were generally less unfavorable with aligners than with fixed appliances, but they were not uniformly unchanged. Targeted subgingival studies commonly found stable or lower periodontal-pathogen burdens and better clinical periodontal indices with aligners (Karkhanechi et al., 2013; Levrini et al., 2015a; Lombardo et al., 2021; Sen et al., 2025). Nevertheless, *Campylobacter rectus* increased in one aligner cohort, and a larger qPCR study found that *Porphyromonas gingivalis*, *Prevotella intermedia*, *Aggregatibacter actinomycetemcomitans*, and *Treponema denticola* increased over time in both appliance groups, although the increases were consistently greater with fixed appliances (Lombardo et al., 2021; Sharma et al., 2025). Under reinforced oral-hygiene conditions, no sustained increase in 11 periodontal marker bacteria was observed during or after clear aligner therapy (Kredig et al., 2025). Sequencing evidence similarly showed stable major subgingival periodontal taxa in some cohorts, while transient *Fusobacterium* and *Prevotella* increases occurred at selected mucosal sites and receded at later time points (Guo et al., 2018; Jiao et al., 2025).

Evidence for opportunistic organisms and health-associated taxa was also inconsistent. Culture of aligners after one day of wear recovered a diverse cultivable community, including *Candida* and periodontal-associated isolates with variable ex vivo biofilm-forming capacity (Baybekov et al., 2023), whereas the pediatric trial described above found a decrease in *C. albicans* (Kapicıi et al., 2025). *Rothia* provides a further example of context-dependent evidence: its relative abundance decreased on the inner aligner surface after 24 h without cleaning, was enriched in saliva from clear aligner users with white spot lesions, and was associated with favorable hygiene indicators in a separate cross-sectional multi-omics study (Yan et al., 2021; Song et al., 2023; Xie et al., 2025a). Thus, no single caries-associated, periodontal, opportunistic, or health-associated taxonomic signature was consistently reproduced across sampling sites and time points.

#### 3.3.3 Microbial functional and metabolic changes

Relative stability in taxonomic diversity does not necessarily imply that microbial function remains unchanged. Alterations in adhesion-related structures, biofilm-forming capacity, nutrient utilization, and metabolite production may occur before substantial changes become detectable in overall community composition. Six studies assessed microbial function or metabolism, but they represented three non-equivalent evidence levels: 16S-based predicted functional profiles, shotgun metagenomic gene-content analysis, and direct metabolomic measurements. One cross-sectional 16S study used PICRUSt to infer Kyoto Encyclopedia of Genes and Genomes (KEGG) pathways and reported appliance-specific differences involving energy, amino acid, and carbohydrate metabolism in the Invisalign group, and membrane transport and nucleotide metabolism in the fixed-appliance group (Wang et al., 2019). These findings represented predicted functions rather than measured genes, transcripts, or metabolites.

Metagenomic sequencing of supragingival plaque from adolescents showed that the overall microbial community and global functional gene profile remained relatively stable during the first 3 months of clear aligner treatment (Wang et al., 2024). Nevertheless, the relative abundances of *Capnocytophaga*, *Neisseria*, and *Arachnia* increased after treatment. Folate biosynthesis, biotin metabolism, and the KEGG pathway annotated as biofilm formation–*Vibrio cholerae* were enriched after 3 months. Genes associated with type IV pili and polar flagella were also more abundant, while functions related to reactive oxygen species increased and starch and sucrose metabolism and galactose metabolism decreased. These shotgun metagenomic findings provided direct gene-level evidence but still reflected functional potential rather than measured metabolic activity.

Microbiome–metabolome analysis of patients with clear aligner-associated white spot lesions revealed a distinct pattern of ecological and metabolic alteration. In this cross-sectional comparison, targeted salivary metabolomics detected 133 metabolites and identified 27 differential candidates, most of which were amino acids or amino-acid-related compounds (Song et al., 2023). The principal enriched pathways included alanine, aspartate and glutamate metabolism; glycine, serine and threonine metabolism; aminoacyl-tRNA biosynthesis; nitrogen metabolism; cyanoamino acid metabolism; and D-glutamine and D-glutamate metabolism. These findings indicate that white spot lesion-associated metabolic alterations extend beyond a simple increase in carbohydrate fermentation and involve broader remodeling of amino acid and nitrogen metabolism.

Three untargeted metabolomic studies further examined metabolic variation across appliance types, oral-health conditions, or plaque compartments. Across six appliance-by-oral-health groups sampled after more than 1 year, both appliance type and oral-health status were associated with plaque-metabolome differences, with pathway categories involving amino acid metabolism, nucleotide metabolism, glycolysis/gluconeogenesis, butanoate metabolism, and cofactor and vitamin metabolism (Xie et al., 2025a). In a longitudinal comparison, clear aligner users showed relatively few within-group metabolite changes, and pathway enrichment was therefore not performed for the within-aligner contrasts; between-appliance comparisons implicated purine, riboflavin, and linoleic acid metabolism (Gong et al., 2025).

Direct comparison of plaque from the aligner-facing interface and the exposed tooth surface has provided further evidence that the aligner creates a metabolically distinct niche. Xie et al. identified 2,502 metabolites, of which amino acids, peptides, and their analogues constituted the largest category, accounting for 20.72% of the detected compounds (Xie et al., 2025b). The metabolomic profiles of aligner-facing and tooth-surface plaque were clearly separated, with generally higher metabolite abundance on tooth surfaces, and the distinction became more pronounced under poor plaque-control conditions. The aligner-facing niche was characterized by lower metabolite diversity and stronger metabolite–metabolite interactions when plaque control was inadequate. The metabolites 4-aminovaleric acid betaine and raffinose were positively associated with plaque index, while integrated multi-omics analysis linked plaque accumulation to amino acid biosynthesis and D-amino acid metabolism. Among 14 differential enzymes involved in D-amino acid metabolism, 13 were more abundant under poor plaque-control conditions.

Local acidity is another dynamic feature of the aligner microenvironment, but its magnitude is strongly influenced by dietary and wearing behavior. These biochemical measurements were considered separately from metabolomic analyses. Inner-aligner fluid pH decreased during 24 h without cleaning, and PETG was associated with a larger transient salivary-pH decrease than TPU in a crossover study (Yan et al., 2021; Gao et al., 2025). Yan et al. found that Coca-Cola consumption caused an immediate reduction in the pH of fluid retained within the aligner and produced pH and community changes that depended on exposure timing (Yan et al., 2026).

Overall, functional and metabolic alterations may be detectable even when conventional measures of microbial diversity remain relatively stable. The available evidence highlights changes in adhesion and biofilm-related functions, amino acid and nitrogen metabolism, metabolite-network organization, and local pH regulation. However, these findings evidence spans predicted pathways, gene-level functional potential, direct metabolite measurements, and biochemical environmental changes and should therefore not be interpreted as a single equivalent evidence layer. Collectively, these findings indicate that clear aligner wear may be accompanied by functional and metabolic changes in addition to taxonomic alterations.

### 3.4 Biofilm control and caries-risk mitigation strategies for clear aligners

During prolonged wear, clear aligners can support biofilm accumulation on their inner surfaces and may alter local ecological conditions. These changes may increase the risk of plaque-associated complications, particularly when oral hygiene is inadequate. However, most intervention studies remain *in vitro* or preclinical, and direct evidence that specific aligner-related interventions reduce the clinical incidence of white spot lesions or dental caries is still limited.

A total of 76 reports contributed to this intervention domain. Based on their primary intervention intent, 19 reports investigated conventional cleaning or decontamination, 33 antimicrobial interface modification, 4 anti-adhesive or antifouling modification, 10 anticaries or remineralizing strategies, and 10 probiotic or environment-responsive strategies. The evidence base was predominantly preclinical: 46 reports used *in vitro* material–microbial models, 7 added ex vivo enamel models, and 9 combined *in vitro* testing with ancillary animal or preliminary human components. The 13 reports were clinical randomized, controlled, nonrandomized, or within-subject interventions, and 1 examined aligners after clinical wear by ex vivo rechallenge. Microbial models ranged from, monospecies cultures to defined polymicrobial biofilms or biofilms derived from saliva or plaque, while treatment duration, exposure frequency, aging protocols, efficacy outcomes, and safety assessments varied markedly. Detailed characteristics of the included intervention studies are summarized in **Supplementary Table S5**.

#### 3.4.1 Conventional cleaning strategies

Cleaning interventions comprised mechanical brushing, chemical immersion, combined mechanical and chemical protocols, and device-assisted approaches such as vibrating baths, ultrasonication, and ozone. Across early within-subject and small comparative studies, water rinsing alone consistently left more deposits than brushing or tablet-assisted brushing, and combining a cleaning agent with vibration or brushing often produced greater removal than a single method (Shpack et al., 2014; Levrini et al., 2015b, 2016; Lombardo et al., 2017). These patterns did not establish a universally superior home protocol: a later fixed-sequence crossover study found declining mutans streptococci and yeast counts over time but no significant differences among five cleaning methods, and a small randomized single-use trial found similar immediate plaque reduction with manual and powered toothbrushes (Harzivartyan et al., 2025; Arslan Çarpar and Yılmaz, 2026).

Chemical and combined protocols generally reduced viable burden or biofilm proxies, but their effects depended on the organism, aligner material, exposure sequence, and endpoint. In a preformed monospecies model, chlorhexidine, 1% sodium hypochlorite, and orthophosphoric acid reduced *S. mutans* below detection, whereas activity against *C. albicans* and the effects of other agents were less uniform (Bonafé et al., 2023). Material-dependent responses were also observed across commercial mouthrinses, and an antiseptic-first sequence followed by brushing was more effective than alternative sequences in an early biofilm model (Alexa et al., 2025; Badnjević et al., 2025). Device-assisted evidence was similarly context specific: ultrasonication with a cationic detergent reduced scanning electron microscopy (SEM)-detected deposits, whereas the 72-h ozone exposure needed for a microbicidal result addressed decontamination of new or manufactured devices rather than routine removal of worn oral biofilm (Lombardo et al., 2017; Nicita et al., 2024). Across these studies, qualitative SEM, crystal-violet staining, adenosine triphosphate (ATP) bioluminescence, colony-forming unit (CFU), metabolic assays, and optical-density readouts measured different aspects of contamination and were not directly interchangeable.

Only a subset of cleaning studies evaluated material consequences together with microbial efficacy. Repeated exposure to several disinfectants caused color changes, and 14 days of brushing or solution-assisted brushing increased surface roughness despite no significant reduction in elastic modulus or ultimate tensile strength (de Silva et al., 2025; Hu et al., 2026). Conversely, a cleaner containing tannic acid and cetylpyridinium chloride showed stain removal and antibacterial activity without evident short-term changes in surface morphology assessed by atomic force microscopy, tensile properties or cell compatibility, but it was not tested under long-term clinical use (Cen et al., 2024). Thus, the available cleaning studies documented mainly immediate or short-term decontamination and did not establish long-term material safety.

#### 3.4.2 Antimicrobial interface modification

The 33 reports assigned primarily to antimicrobial interface modification included surface-deposited coatings, antimicrobial agents incorporated into thermoformed or directly printed aligner matrices, modified attachment resins or primers, and laser-created structures used to retain antimicrobial nanoparticles. The active platforms comprised contact-active cationic polymers and quaternary ammonium chemistries, chitosan-based systems, N-halamines, metal and metal oxide nanoparticles, photocatalytic and metal–organic framework constructs, and release-active organic agents. Most studies used *S. mutans* monospecies models; smaller subsets evaluated *P. gingivalis*, *C. albicans*, *F. nucleatum*, other indicator organisms, or polymicrobial and saliva-derived biofilms. The reported endpoints included planktonic growth, surface attachment, viable counts, metabolic activity, acidogenicity, and biofilm biomass, so efficacy estimates obtained with different models and assays were not treated as directly comparable.

Contact-active and polymer-based modifications generally reduced early attachment or subsequent biofilm formation, although the magnitude depended on formulation and endpoint. A crosslinked carboxymethylcellulose–chitosan coating on PETG reduced the measured bacterial burden by approximately 75% (Park et al., 2018), whereas quaternary-ammonium-functionalized gold nanoclusters inhibited *S. mutans* attachment and biofilm formation (Xie et al., 2020). Incorporating chitosan nanoparticles into a directly printed resin produced a concentration-dependent pattern, with 3% and 5% formulations reducing *S. mutans* colonies more clearly than the 2% formulation (Taher and Rasheed, 2023). Release-active cellulose and attachment-resin systems produced related but methodologically distinct effects: a cellulose material loaded with essential oils delayed *S. mutans* and *S. mitis* biofilm development under both static and simulated salivary flow conditions, while a chlorhexidine-containing attachment resin maintained reductions in *S. mutans* viable counts, metabolic activity, and lactic acid production after one month of aging (Astasov-Frauenhoffer et al., 2023; Cao et al., 2023). Broader contact-active systems also showed activity against multiple organisms: an N-halamine interface reduced *S. mutans*, *C. albicans*, and *F. nucleatum* biofilms (Zhou et al., 2025), and a colorless quaternized tannic acid coating achieved greater than 99% killing of *S. mutans* and approximately 82.9% killing of *C. albicans*, with inhibition retained in a polymicrobial model after saliva-related challenges (Chen et al., 2026a).

Metal-based, metal oxide-based, and light-activated systems frequently showed strong antibacterial effects, but these were organism-, dose-, and activation-dependent. ZnO coatings suppressed *S. mutans* over seven- or 15-day observation periods (Anita et al., 2023), whereas inhibition of *C. albicans* was minimal or not clearly demonstrated in the same studies (Batool et al., 2026). Combined ZnO–MgO produced greater inhibition of *S. mutans* and *Lactobacillus* than either oxide alone (Gharibnavaz et al., 2025), while ultraviolet A (UVA)-activated TiO_2_ reduced *S. mutans* biomass and *gtfB* expression in a dose-dependent manner (Pourhajibagher et al., 2025). A visible-light-responsive TiO_2_–Cu_2_O–Cu coating reported 99.35%–99.89% antibacterial activity against *S. mutans* under the tested conditions (Ha et al., 2024). Silver-containing approaches likewise affected both planktonic and sessile bacteria: a 2% silver electrospun TPU membrane reduced planktonic *S. mutans* by approximately 5–6 log units and biofilm metabolic activity by about 44% (Zhang et al., 2025), whereas PETG containing 10% silver produced an approximately eight-log reduction and suppressed culture acidification (Huang et al., 2025). In newer composite interfaces, a silver-containing porphyrinic metal–organic framework reduced biofilm biomass by approximately 72.2%, and a laser-generated porous micro/nanostructure loaded with silver nanoparticles yielded approximately 62.9% *S. mutans* inhibition (Li et al., 2026b; Sang et al., 2026).

#### 3.4.3 Anti-adhesive and antifouling interface modification

Anti-adhesive and antifouling strategies differ from conventional antimicrobial strategies in that their core aim is not to kill bacteria directly. Instead, they provide protection by reducing protein adsorption, decreasing initial bacterial adhesion, and inhibiting early biofilm formation. Three studies used zwitterionic hydration layers. Carboxybetaine modification reduced protein adsorption and a pooled-saliva multispecies biofilm, polySBMA improved aqueous lubrication while reducing protein and bacterial attachment, and incorporation of MPC reduced protein adsorption and downstream *S. mutans* biofilm without major mechanical impairment (Wu et al., 2024; Paik et al., 2025; Wei et al., 2025). A separate biomimetic strategy used shark-skin-like microtopography to reduce the effective contact area and *S. mutans* attachment without an incorporated antimicrobial agent (Zhu et al., 2025).

Accordingly, lower CFU or biomass in these studies was interpreted as a downstream consequence of reduced colonization, not as direct antimicrobial activity. Evidence was strongest for protein adsorption, early attachment, and short-term biofilm formation. The available aging assessments lasted seven days for carboxybetaine-treated resin and two weeks for polySBMA, while long-term brushing, repeated insertion and removal, retention after thermoforming, and control of established mature biofilm were not systematically evaluated (Wu et al., 2024; Wei et al., 2025).

#### 3.4.4 Anticaries and remineralizing functional strategies

Caries-preventive strategies associated with clear aligners should not be limited to reducing bacterial numbers, but should also address the balance between enamel demineralization and remineralization. The occurrence of white spot lesions is closely related to local plaque accumulation, enhanced acidogenic metabolism, pH reduction, and mineral loss. Because clear aligners cover tooth surfaces and may reduce salivary clearance and prolong the retention of plaque and fermentable substrates, attachment margins, interproximal surfaces, and gingival margins may be particularly susceptible to demineralization. Accordingly, materials with dual antimicrobial and remineralizing functions have gradually attracted attention.

Caries-risk-mitigation studies were included when a clear-aligner-specific preventive or remineralizing intervention was evaluated against a mineral, lesion, ion-release, acidogenic, or related anticaries outcome. The strategies comprised fluoride- or calcium/phosphate-releasing aligner surfaces, remineralizing or bioactive attachment materials, pH-responsive antimicrobial–mineral systems, and topical enamel-protective regimens.

Direct aligner-surface functionalization provided mainly *in vitro* or ex vivo evidence. A fluoride-coated aligner released fluoride for 14 days, could be recharged, reduced *S. mutans* activity, and limited enamel mineral loss in demineralization and pH-cycling models (Yan et al., 2023). A pH-responsive coating incorporating tannic acid–copper, zwitterionic, and calcium phosphate (CaP) components combined antibiofilm activity under acidic conditions with hydroxyapatite-like deposition on demineralized enamel, while a ZIF-8@nHA system promoted a more oriented enamel-like remineralized layer in an acid-etched model (Qin et al., 2024; Liang et al., 2025). Piezoelectric ZnO–PVDF functionalization also reduced *in vitro* demineralization when mechanically activated, but increased haze at higher ZnO loadings (Ye et al., 2025).

Attachment-based approaches used bioactive glass, bredigite, ion-releasing restorative materials, or combined quaternary-ammonium and nano-amorphous-calcium-phosphate resins. Several reduced lesion depth or mineral loss and retained clinically relevant bond strength in short ex vivo or *in vitro* models (Jiang et al., 2023; Kölüş et al., 2025; Petapa et al., 2025). DMADDM–NACP formulations additionally reduced *S. mutans* viability, metabolism, and acid production and released Ca/P ions, but direct enamel demineralization or remineralization was not measured in those formulation studies (Alqarni et al., 2025, 2026). Clinical evidence remained limited: a small six-month controlled trial reported improved attachment-adjacent DIAGNOdent values with CPP-ACP, nano-hydroxyapatite, or fluoride varnish, whereas a 10-patient study of a fluoride and essential oil spray study assessed salivary fluoride and qualitative deposits (Cremonini et al., 2025; Ünal et al., 2025). None of these studies established a reduction in incident white spot lesions or dental caries as a definitive clinical outcome.

#### 3.4.5 Probiotic and environment-responsive strategies

Probiotic ecological modulation and environment-responsive antimicrobial systems were treated as two distinct intervention concepts. The former aimed to influencemicrobial competition or activity, whereas the latter used local acidity, mechanical loading, or a sensed disease-related signal to activate antimicrobial action.

Probiotic studies included an *in vitro Streptococcus salivarius* candidate and its cell-free supernatant, which suppressed *S. mutans* in mono-, dual-, and plaque-derived multispecies biofilms on orthodontic surfaces *in vitro Streptococcus salivarius* (Gong et al., 2023). In clinical studies, short probiotic regimens reduced aligner-biofilm CFU and extracellular polymeric substance (EPS) signals, plaque and bleeding scores, or volatile sulfur compounds (Lanzetti et al., 2025; Thakur et al., 2026; Vasoglou et al., 2026). However, the trials evaluating plaque and bleeding outcomes or volatile sulfur compounds did not include microbial community profiling, and the crossover biofilm trial lasted seven days per formulation. These outcomes documented short-term clinical or biofilm changes but did not establish sustained changes in microbial community structure or function.

Environment-responsive systems included pH-switchable peptide or antimicrobial–mineral coatings, mechanically activated piezoelectric materials, and a wireless device coupling pH and nitrite sensing with on-demand drug release. The pH-responsive coatings suppressed *S. mutans* under acidic conditions, and one peptide platform designed using machine learning also reduced caries in an animal model while shifting the measured community away from potentially pathogenic genera and toward health-associated genera (Sun et al., 2022; Qin et al., 2024; Li et al., 2026b). Piezoelectric systems showed stronger antibacterial activity under mechanical activation, and the wireless platform reduced pathogen burden and tissue injury in preclinical caries and periodontal models (Shi et al., 2023; Ye et al., 2025; Zhang et al., 2026). These findings demonstrated conditionally activated antimicrobial action. Apart from the preclinical peptide study, they did not directly establish selective preservation of commensal communities.

#### 3.4.6 Durability, performance, and safety

Across intervention classes, durability was evaluated with non-equivalent protocols, including storage, artificial-saliva or enzyme immersion, repeated washing or cleaning, abrasion or brushing, bending or chewing simulation, thermoforming, and short animal or clinical wear. Among antimicrobial interfaces, quaternary-ammonium-modified gold nanoclusters retained activity after at least three months of storage and more than three reuse cycles, while a polysilsesquioxane coating remained active after chemical and mechanical challenges and two weeks of canine intraoral wear (Xie et al., 2020; Park et al., 2022). A visible-light-responsive TiO_2_–Cu_2_O–Cu coating retained its tested antibacterial and material properties after 14 days in artificial saliva, scratch testing, and 10,000 bending cycles, and a quaternized tannic acid coating retained polymicrobial inhibition after artificial-saliva exposure and three months of storage (Ha et al., 2024; Chen et al., 2026a).

Other durability tests identified progressive loss rather than stable activity. Thermoforming and aging reduced the thickness and antibiofilm benefit of a chitosan-containing nanocoating, and only approximately 60%–65% of a ZnO–MgO coating remained after artificial-saliva agitation and simulated brushing (Krishnakumaran et al., 2023; Gharibnavaz et al., 2025). An N-halamine interface survived several storage and mechanical challenges, but its antibacterial activity declined during continued artificial-saliva washing and no clear colony reduction remained at about day 15 (Zhou et al., 2025). Repeated conventional maintenance could also modify the underlying material: 14 days of brushing or solution-assisted brushing increased surface roughness, and repeated exposure to several disinfectants produced detectable color changes (de Silva et al., 2025; Hu et al., 2026). These protocols demonstrated strategy-specific persistence or degradation but were insufficiently standardized to estimate clinical service life.

Material-performance effects were formulation dependent. The TiO_2_–Cu_2_O–Cu coating increased hardness from about 240 to 379 MPa while retaining flexibility and transparency. Porphyrinic metal–organic framework interfaces, silver-loaded micro/nanostructured interfaces, and quaternized tannic acid interfaces also generally preserved the principal mechanical and optical properties measured under their respective test conditions (Ha et al., 2024; Chen et al., 2026a; Li et al., 2026a; Sang et al., 2026). In contrast, the 4% TiO_2_ formulation produced the strongest tested antibacterial effect but markedly reduced transparency, whereas 2% TiO_2_ provided a more favorable efficacy–esthetic balance (Pourhajibagher et al., 2025). Combined ZnO–MgO was more antibacterial but less optically favorable than ZnO alone, and higher chitosan, silver, or chlorhexidine-containing filler levels altered translucency, polymerization, elongation or failure-related properties, or color (Cao et al., 2023; Taher and Rasheed, 2023; Gharibnavaz et al., 2025; Zhang et al., 2025; Azeez and Mudhir, 2026). Thus, the formulation giving the largest microbial reduction was not necessarily the formulation with the most favorable overall material profile.

Safety evidence consisted mainly of *in vitro* cell-viability or cytocompatibility assays, sometimes supplemented by hemolysis or general animal observations. Cell-viability and live/dead assays generally detected no significant cytotoxicity for the tested gold-based, polysilsesquioxane, antimicrobial-peptide, fluoride, Ag-, Cu-, metal-oxide-, N-halamine-, MOF-, and antifouling interfaces, with viability commonly remaining above 80%–90% under the reported conditions (Xie et al., 2020; Park et al., 2022; Sun et al., 2022; Yan et al., 2023; Ha et al., 2024; Wu et al., 2024; Zhang et al., 2025; Zhou et al., 2025; Chen et al., 2026a; Sang et al., 2026). However, several formulations showed exposure-dependent effects. Essential-oil-loaded cellulose reduced gingival-fibroblast viability to 49% without rinsing, although viability recovered to approximately 72%–75% after prolonged rinsing (Astasov-Frauenhoffer et al., 2023). Addition of zwitterionic and CaP layers reduced cell viability from approximately 92% to 85% and 81%, respectively (Qin et al., 2024), while 2% chitosan resin produced 85.8% viability in 3T3 cells despite no significant overall between-group difference (Taher and Rasheed, 2023). The cationic P11 cleaner caused significant viability loss after 24-h exposure at 250–500 μg/mL, demonstrating clear concentration- and time-dependent cytotoxicity (Seo et al., 2026).

Hemolysis remained absent or below 2% for several gold-based, TZC, QTA, SiO_2_, and N-halamine systems, whereas selenium nanoparticles were assessed only by brine shrimp lethality and showed lower toxicity at lower concentrations (Xie et al., 2020; Zhang et al., 2020; Qin et al., 2024; Dawood et al., 2025; Zhou et al., 2025; Chen et al., 2026b). Animal and short clinical assessments generally reported no evident treatment-related abnormalities or adverse reactions (Xie et al., 2020; Zhang et al., 2020, 2026; Wei et al., 2025; Chen et al., 2026a; Yu et al., 2026). Direct evidence regarding oral mucosal irritation remained limited, because safety assessments were dominated by *in vitro* cytocompatibility and hemolysis assays, with only limited dedicated irritation or short-term preclinical and clinical observations.

## 4 Discussion

The findings of this scoping review are organized across three evidence domains. **Figure 3** provides a visual synthesis of all three domains. Panels A and B summarize the material, manufacturing, and surface attributes examined in interface studies and situate these attributes within the clear aligner–tooth interface. Panel C summarizes the compartment- and time-dependent changes in oral microbial ecology reported during clear aligner wear. Panel D organizes the intervention evidence into five strategy categories: conventional cleaning, antimicrobial interface modification, anti-adhesive and antifouling interface modification, anticaries and remineralizing approaches, and probiotic and environment-responsive strategies.hemicConsidered together, the three evidence domains provide complementary but non-interchangeable levels of evidence. Interface studies identify material, topographical, and exposure-related conditions that may influence microbial adhesion, but these findings do not directly predict ecological responses during clinical wear. Clinical studies further indicate that microbial responses vary by oral compartment and time. Most intervention studies, however, assess short-term changes in microbial burden, biofilm biomass, acidogenicity, or mineral loss rather than changes in community structure or function or patient-level disease outcomes. This cross-domain synthesis reveals a recurring disconnect between mechanistic plausibility, localized ecological response, and demonstrated clinical benefit. Accordingly, *in vitro* antimicrobial performance alone is insufficient for ranking materials or preventive strategies.

**Figure 3:**
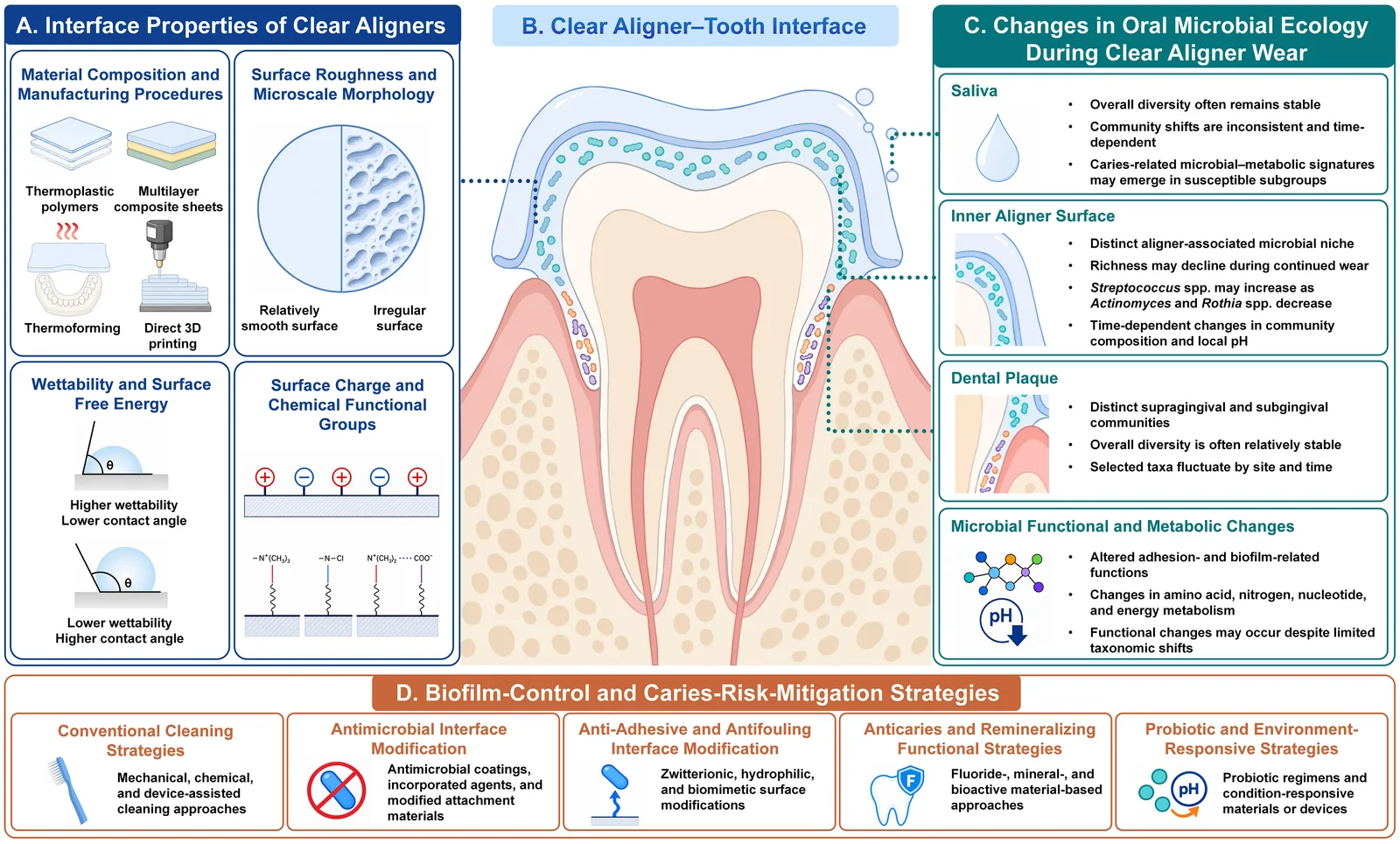
Clear aligner interface properties, oral microbial ecology during wear, and biofilm-control and caries-risk-mitigation strategies. **(A)** Major material and interfacial attributes of clear aligners, including material composition and manufacturing procedures, surface roughness and microscale morphology, wettability and surface free energy, and surface charge and chemical functional groups. **(B)** Schematic representation of the clear aligner–tooth interface and microbial accumulation at the inner aligner surface and adjacent dental and gingival sites. **(C)** Reported changes in oral microbial ecology during clear aligner wear, encompassing saliva, the inner aligner surface, dental plaque, and microbial functional and metabolic profiles. **(D)** Biofilm-control and caries-risk-mitigation strategies, classified as conventional cleaning, antimicrobial interface modification, anti-adhesive and antifouling interface modification, anticaries and remineralizing approaches, and probiotic and environment-responsive strategies.

The interface evidence does not support a simple model in which a particular polymer or single surface parameter predicts biofilm accumulation. PETG, TPU, multilayer sheets, and directly printed resins differed in roughness, wettability, morphology, chemistry, and aging behavior. However, these broad material categories did not consistently predict microbial adhesion (Tektas et al., 2020; Chen et al., 2025; Martínez Gil-Ortega et al., 2025). Local surface geometry may be particularly relevant. Recessed, folded, scratched, or sheltered regions frequently retained more biofilm than flatter and more exposed areas (Low et al., 2011; Rodríguez Fernández et al., 2024; de Souza Schacher et al., 2025; Mota et al., 2026). This pattern was not consistently captured by mean roughness measurements. Moreover, microorganisms encounter a saliva-conditioned surface rather than the chemically pristine polymer. Studies of other dental materials indicate that salivary pellicle formation can alter measured surface energetics and may reduce the explanatory value of bare-surface properties for bacterial attachment (Sang et al., 2020). Roughness, wettability, surface free energy, chemistry, and charge should therefore be interpreted as interrelated characteristics rather than independent predictors. Their concurrent modification may also help explain why observed microbial effects cannot usually be attributed to a single measured surface property.

Experimental model selection also determines which aspects of interface–biofilm interactions can be evaluated. Static monospecies assays are useful for screening intrinsic material effects and identifying dose-response relationships. However, they exclude interspecies competition, salivary flow, repeated nutrient exposure, pellicle renewal, and host-related factors. Defined multispecies and saliva- or plaque-derived microcosm models offer greater ecological complexity. Dynamic-flow systems can additionally evaluate microbial retention under fluid movement. Nevertheless, model complexity alone does not ensure clinical relevance unless the system remains reproducible and corresponds to the research question. Validated oral microcosm models outside the clear-aligner literature show that donor origin and sucrose exposure can influence community organization and cariogenic behavior under controlled conditions (Rudney et al., 2012). Tooth-on-a-chip and gingival-crevice-on-chip studies also illustrate possible approaches for integrating biomaterials, fluid flow, host tissues, and microbial exposure. These platforms have not yet been validated for clear-aligner-specific applications (França et al., 2020; Makkar et al., 2023). Aging represents another source of variation. Clinically exposed aligners may undergo changes in roughness, porosity, chemistry, and morphology. Brushing, cleaning, and exposure to cigarette smoke may further modify the interface (Lira et al., 2023; Eslami et al., 2024; Galisteu-Luiz et al., 2026; Hu et al., 2026). Findings from pristine specimens and short static incubations should therefore be treated as mechanistic or screening evidence, not as direct estimates of biofilm behavior during clinical wear.

The clinical microbiological evidence is more consistent with localized and time-dependent responses than with a uniform and sustained whole-mouth shift. Alpha diversity remained relatively stable in several studies of saliva, supragingival plaque, and subgingival plaque. More dynamic changes were sometimes observed on the inner aligner surface or at selected mucosal sites (Yan et al., 2021; Rouzi et al., 2023; Gao et al., 2025; Jiao et al., 2025). Multisite studies also indicated that variation among oral compartments could exceed variation associated with treatment time or appliance group (Duong et al., 2022; Peregrina et al., 2025). This finding is consistent with the established biogeography of the oral cavity. Different oral surfaces support distinct microbial communities, and saliva does not necessarily represent tooth-associated or subgingival plaque (Simon-Soro et al., 2013). Apparent disagreement among clear-aligner studies may therefore arise partly from differences in sampling compartment, anatomical site, follow-up schedule, or analytical method. Comparisons are most informative when these methodological dimensions are aligned.

Taxonomic and functional findings also represent different levels of evidence. Changes in relative abundance do not necessarily indicate changes in absolute microbial burden, metabolic activity, or disease risk. Similarly, 16S-based pathway prediction estimates functional potential from taxonomic profiles, whereas shotgun metagenomics characterizes microbial gene content. Metabolomics directly measures chemical compounds but may not establish whether individual metabolites are microbial, host-derived, or jointly produced. The included studies reported differences involving amino acid, nitrogen, nucleotide, carbohydrate, and energy metabolism, together with changes in local acidity (Wang et al., 2019; Song et al., 2023; Gong et al., 2025; Xie et al., 2025b, 2025a). These signals were obtained from different populations, compartments, time points, and analytical workflows. They have not been prospectively validated as clear-aligner-specific biomarkers. Accordingly, these microbial–metabolic patterns should currently be regarded as candidate early-monitoring signals that may complement, rather than replace, conventional visual assessment of white spot lesions and plaque indices. Outside the clear-aligner evidence set, longitudinal studies suggest that microbial profiles may precede caries development in young children or WSL development during fixed-appliance treatment (Blostein et al., 2022; Cheng et al., 2026). These studies illustrate the potential feasibility of microbiome-informed risk prediction.

The intervention evidence was extensive but uneven in translational maturity. Conventional cleaning studies included several clinical or within-subject evaluations, although most assessed immediate or short-term changes in deposits, CFU, ATP, or other biofilm-related outcomes. Antimicrobial interface modifications were predominantly tested *in vitro*, frequently using monospecies *S. mutans* models. Anticaries and remineralizing systems relied mainly on material, pH-cycling, or ex vivo enamel models. These studies provide proof-of-concept or mechanistic evidence, but they do not permit reliable ranking of the interventions. Microbial models, exposure schedules, comparators, and outcome measures differed substantially. Reductions in planktonic growth, initial attachment, established biomass, acid production, and enamel mineral loss represent different intervention targets and should not be combined into a single efficacy scale. Clinical translation would require reproducible microbial or mineral effects, persistence under relevant aging and maintenance conditions, preservation of material performance, and confirmation of patient-level benefit. Several cleaning, coating, probiotic, fluoride-releasing, and bioactive-attachment strategies produced promising findings (Shpack et al., 2014; Levrini et al., 2015b; Xie et al., 2020; Park et al., 2022; Yan et al., 2023; Qin et al., 2024; Ünal et al., 2025). However, no intervention was sufficiently supported across this full translational sequence to justify its designation as a universally superior strategy.

The intervention findings also distinguish between reducing microbial quantity and modifying an unfavorable microbial ecology. Broad antimicrobial approaches can markedly reduce viable microorganisms. However, the largest reduction may not represent the most favorable ecological outcome if health-associated organisms or beneficial microbial functions are also affected. External human and complex-biofilm studies of chlorhexidine reported changes in microbial composition, salivary acidity, metabolic activity, and post-treatment regrowth despite an initial reduction in microbial burden (Bescos et al., 2020; Chatzigiannidou et al., 2020). These findings should not be directly extrapolated to immobilized or low-release aligner coatings. They nevertheless illustrate why microbial killing alone provides an incomplete assessment of ecological effects. Anti-adhesive and antifouling interfaces use a different approach by reducing protein adsorption or initial attachment without necessarily applying continuous bactericidal pressure (Wu et al., 2024; Paik et al., 2025; Wei et al., 2025). Probiotic interventions aim to modify microbial competition, whereas environment-responsive systems aim to activate or enhance antimicrobial action under selected local conditions (Sun et al., 2022; Gong et al., 2023; Qin et al., 2024; Li et al., 2026b). A pH-responsive peptide has also suppressed cariogenic traits while increasing commensal competitiveness in a non-aligner dual-species model (Jiang et al., 2023). However, most clear-aligner studies did not directly evaluate preservation of health-associated communities or microbial functions. Microbiome selectivity should therefore not be inferred solely from pH responsiveness, antifouling activity, or incomplete microbial suppression.

Durability, material performance, and safety are integral components of intervention efficacy. Some modified interfaces retained antimicrobial activity after storage, exposure to artificial saliva, washing, bending, or mechanical challenge. Others showed coating loss, reduced activity, or altered surface properties after thermoforming, aging, or simulated cleaning (Xie et al., 2020; Park et al., 2022; Krishnakumaran et al., 2023; Ha et al., 2024; Gharibnavaz et al., 2025; Zhou et al., 2025; Chen et al., 2026b). The protocols were too heterogeneous to define a common estimate of clinical service life. Material trade-offs were also formulation dependent. Increasing the concentration of an antimicrobial component sometimes improved microbial inhibition while impairing transparency, translucency, elongation, polymerization, or color stability (Cao et al., 2023; Taher and Rasheed, 2023; Pourhajibagher et al., 2025; Zhang et al., 2025; Azeez and Mudhir, 2026). Under the reported assay conditions, several interfaces based on gold, polysilsesquioxane, antimicrobial peptides, fluoride, metals, metal oxides, N-halamines, metal–organic frameworks, or antifouling chemistries showed favorable cytocompatibility. In contrast, essential-oil-loaded cellulose, layered zwitterionic/CaP interfaces, chitosan-containing resin, and the P11 cleaner showed formulation-, dose-, or exposure-dependent reductions in cell viability (Astasov-Frauenhoffer et al., 2023; Taher and Rasheed, 2023; Qin et al., 2024; Seo et al., 2026). These assays establish compatibility only under their respective experimental conditions. They do not independently establish long-term biological tolerance during repeated clinical wear. Translation therefore requires a balance among antimicrobial or antifouling efficacy, durability, optical acceptability, mechanical integrity, controlled release, and safety under clinically relevant exposure conditions. Accordingly, favorable *in vitro* cytocompatibility should not be interpreted as evidence of the absence of oral mucosal irritation during prolonged clinical use.

The clinical relevance of clear-aligner-associated microbial changes is likely to depend on interactions among the appliance, patient characteristics, and behavioral exposures. Age, dentition stage, baseline plaque control, diet, salivary conditions, smoking, treatment duration, attachment configuration, and cleaning adherence may modify the observed response. Mixed-dentition cohorts, adolescents with poor plaque control, smokers, and patients with existing cariogenic or periodontal susceptibility should not be assumed to respond identically to healthy adults with effective oral hygiene (Chen et al., 2024; Kapicıi et al., 2025; Xie et al., 2025a, 2025b; Galisteu-Luiz et al., 2026). Evidence linking smoking with a pathogen-enriched and commensal-depleted subgingival microbiome further supports considering tobacco exposure as a relevant ecological modifier (Mason et al., 2015). Pending stronger clinical intervention evidence, a cautious approach is to reinforce oral and aligner hygiene, limit repeated exposure to fermentable carbohydrates and acidic substances during wear, and encourage smoking cessation. Fluoride or remineralizing measures may also be considered when clinically indicated.

## 5 Future prospects

Further clinical research would benefit from multicenter longitudinal designs, more consistent sampling procedures, and longer follow-up. Greater comparability may be achieved by harmonizing the sampled oral compartments, principal sampling time points, microbiological outcomes, and reporting of relevant clinical and behavioral variables. Such developments could reduce the uncertainty arising from the small samples and short observation periods that characterize much of the current evidence and may provide a stronger basis for examining whether clear aligner–associated microbial changes persist or translate into adverse clinical outcomes.

Experimental research may progressively extend beyond static monospecies assays toward models that more closely reproduce intraoral conditions. Multispecies or saliva-derived microcosms could better represent microbial interactions, whereas dynamic systems incorporating salivary flow, nutrient fluctuations, pH cycling, and repeated material exposure may improve the clinical relevance of biofilm testing. Organ-on-chip and tissue-integrated platforms may further support investigation of host–material–microbial interactions that cannot be adequately represented by conventional culture models. Existing oral microcosm and tooth- or gingival-crevice-on-chip platforms provide conceptual foundations for these developments, although their application to clear aligners remains limited (Rudney et al., 2012; França et al., 2020; Makkar et al., 2023). Future material studies should also incorporate standardized intraoral or clinically relevant simulated aging pretreatment before microbial evaluation. Aging protocols should clearly define clinically relevant parameters such as exposure duration, salivary conditioning, thermal and pH cycling, mechanical wear, and repeated cleaning, followed by surface characterization and microbial or biofilm assessment.

Future material development could combine antimicrobial coatings, anti-adhesive surfaces, and environment-responsive systems with a stronger emphasis on ecological compatibility. Rather than pursuing indiscriminate microbial suppression, these approaches may aim to restrict disease-associated adhesion and biofilm activity while limiting disturbance of health-associated microbial communities. pH-responsive antimicrobial systems illustrate one possible route toward conditionally activated activity (Jiang et al., 2023), although their selectivity within complex oral communities remains to be established. Joint assessment of microbial effects, durability, optical and mechanical properties, and biocompatibility may help identify strategies with greater translational potential.

The integration of metagenomics, metabolomics, and proteomics with longitudinal clinical data may support individualized risk assessment. Linking multi-omics profiles with local pH, enamel mineral changes, periodontal measures, treatment characteristics, and behavioral exposures could help identify candidate microbial or metabolic signatures of susceptibility. Prospective studies should determine whether these signatures precede clinically visible white spot lesions and establish their reproducibility and predictive value. If validated, they may support risk-stratified preventive strategies and guide the selection of cleaning, remineralizing, or material-based interventions.

## 6 Limitations

The breadth of this scoping review also limits the inferences that can be drawn from the assembled evidence. The included studies varied substantially in aligner materials, manufacturing procedures, microbial models, sampling compartments, follow-up schedules, analytical methods, interventions, and outcome definitions. Culture, targeted molecular assays, sequencing, metabolomics, and material-characterization methods also represented different levels of evidence and were not directly interchangeable. This heterogeneity precluded quantitative synthesis and direct comparison across studies. Accordingly, the evidence maps and narrative synthesis describe the distribution and general patterns of the literature but do not provide pooled effect estimates, establish causal relationships between interface properties, microbial changes, and clinical disease, or support definitive ranking of materials or interventions.

Although the search covered three major databases, included supplementary citation searching, and imposed no language restrictions, potentially relevant evidence may still have been missed. Gray literature and conference abstracts without sufficient methodological and outcome data were excluded, and two reports identified for full-text assessment could not be retrieved. These factors may have introduced retrieval or publication bias. In addition, the search reflects evidence available up to August 31, 2026, and may not capture subsequent developments in this rapidly evolving field. Formal risk-of-bias assessment and certainty-of-evidence grading were not undertaken because the review was designed primarily to map the scope and characteristics of the evidence.

Some reports contributed evidence to more than one domain, and classification required judgment when interface characteristics, microbial outcomes, and preventive interventions were examined within the same study. Cross-classification was based on the research question and the evidence provided, but the boundaries between domains were not always absolute; domain-specific counts should therefore not be interpreted as independent study totals. More broadly, much of the evidence was derived from short-term *in vitro* or preclinical models, while clinical studies frequently involved limited samples, heterogeneous sampling strategies, and surrogate microbial or mineral outcomes. Evidence directly linking the observed interface or microecological changes to incident white spot lesions, caries, periodontal deterioration, or sustained clinical benefit remains limited. These constraints reduce the generalizability of the findings and restrict conclusions regarding long-term clinical effectiveness and safety.

## 7 Conclusion

This scoping review mapped 132 reports across three interrelated domains: clear aligner interface properties and microbial adhesion, oral microbial ecology during clear aligner wear, and biofilm-control and caries-risk-mitigation strategies. The available evidence indicates that microbial colonization of clear aligners is shaped by interacting material, surface, environmental, and experimental factors rather than by a single polymer, manufacturing method, or interface parameter. During clinical wear, microbial responses appear to be compartment- and time-dependent. Salivary and tooth-associated communities often remain relatively stable at the community level, whereas the inner aligner surface may form a distinct microbial niche exhibiting localized taxonomic, functional, metabolic, and pH-related changes. A diverse range of cleaning, antimicrobial, anti-adhesive, remineralizing, probiotic, and environment-responsive strategies has been investigated. However, much of this evidence remains short-term and preclinical, and no intervention can currently be regarded as universally superior or as definitively reducing white spot lesions, caries, or periodontal deterioration. Overall, the evidence does not support viewing clear aligners as either microbiologically inert or uniformly disruptive to the oral microbiota. Their biological effects are more appropriately understood as context-dependent interactions among the material interface, oral compartment, duration of exposure, patient susceptibility, and preventive practices. More standardized and clinically representative evidence may help translate this framework into reliable risk assessment and preventive management during clear aligner treatment.
