# Supplementary material for: Clear aligner interface properties, oral microbial ecology, and strategies for biofilm control and caries-risk mitigation: a scoping review
Source: Front Cell Infect Microbiol. 2026 Oct 23;16:1951083. doi: 10.3389/fcimb.2026.1951083 (PMC13741175; doi:10.3389/fcimb.2026.1951083)
Supplement: Supplementary file 1 [file Table1.docx]

**Supplementary Table S1. PRISMA-ScR Checklist**

| **SECTION / TOPIC** | **#** | **CHECKLIST ITEM** | **REPORTED ON (Section / Page)** |
| --- | --- | --- | --- |
| Title | 1 | Identify the report as a scoping review. | Title page (page 1) |
| Abstract | 2 | Provide a structured summary that includes (as applicable) background, objectives, eligibility criteria, sources of evidence, charting methods, results, and conclusions that relate to the review questions and objectives. | Abstract (pages 1–2) |
| Rationale | 3 | Describe the rationale for the review in the context of what is already known. | Introduction (pages 2–3) |
| Objectives | 4 | Provide an explicit statement of the questions and objectives being addressed with reference to their key elements (e.g., population or participants, concepts, and context) or other relevant key elements used to conceptualize the review questions and objectives. | Introduction and Methods section 2.1 — PCC framework (page 3) |
| Protocol and registration | 5 | Indicate whether a review protocol exists; state if and where it can be accessed (e.g., a Web address); and if available, provide registration information, including the registration number. | Methods section 2.1 — review not prospectively registered (page 3) |
| Eligibility criteria | 6 | Specify characteristics of the sources of evidence used as eligibility criteria (e.g., years considered, language, and publication status), and provide a rationale. | Methods sections 2.1 and 2.3 — PCC framework and eligibility criteria (pages 3–4) |
| Information sources | 7 | Describe all information sources in the search (e.g., databases with dates of coverage and contact with authors to identify additional sources), as well as the date the most recent search was executed. | Methods section 2.2 — PubMed, Embase, Web of Science Core Collection, supplementary citation searching, and latest search date (page 3) |
| Search | 8 | Present the full electronic search strategy for at least 1 database, including any limits used, such that it could be repeated. | Methods section 2.2 and Supplementary Table S2 — complete database-specific search strategies, search dates, and record counts (page 3) |
| Selection of sources of evidence | 9 | State the process for selecting sources of evidence (i.e., screening and eligibility) included in the scoping review. | Methods section 2.4 — independent title/abstract and full-text screening by two authors, with disagreements resolved by consensus (page 4) |
| Data charting process | 10 | Describe the methods of charting data from the included sources of evidence (e.g., calibrated forms or forms that have been tested by the team before their use, and whether data charting was done independently or in duplicate) and any processes for obtaining and confirming data from investigators. | Methods section 2.5 — two authors participated in data charting and verification; uncertainties or discrepancies were resolved through discussion (pages 4–5) |
| Data items | 11 | List and define all variables for which data were sought and any assumptions and simplifications made. | Methods section 2.5 and Supplementary Tables S3–S5 (pages 4–5) |
| Critical appraisal of individual sources of evidence | 12 | If done, provide a rationale for conducting a critical appraisal of included sources of evidence; describe the methods used and how this information was used in any data synthesis (if appropriate). | Not applicable — formal risk-of-bias assessment and certainty-of-evidence grading were not undertaken; rationale reported in Limitations (page 21) |
| Synthesis of results | 13 | Describe the methods of handling and summarizing the data that were charted. | Methods section 2.5 — data were tabulated and summarized descriptively within three predefined evidence domains; no quantitative meta-analysis was undertaken (page 5) |
| Selection of sources of evidence | 14 | Give numbers of sources of evidence screened, assessed for eligibility, and included in the review, with reasons for exclusions at each stage, ideally using a flow diagram. | Results section 3.1 and Figure 1 (pages 5 and 34) |
| Characteristics of sources of evidence | 15 | For each source of evidence, present characteristics for which data were charted and provide the citations. | Results sections 3.2–3.4 and Supplementary Tables S3–S5 (pages 5–17) |
| Critical appraisal within sources of evidence | 16 | If done, present data on critical appraisal of included sources of evidence (see item 12). | Not applicable — formal critical appraisal was not performed (see item 12) |
| Results of individual sources of evidence | 17 | For each included source of evidence, present the relevant data that were charted that relate to the review questions and objectives. | Results sections 3.2–3.4 and Supplementary Tables S3–S5 (pages 5–17) |
| Synthesis of results | 18 | Summarize and/or present the charting results as they relate to the review questions and objectives. | Results sections 3.2–3.4, Figures 2–3, and Supplementary Tables S3–S5 (pages 5–17 and 35–36) |
| Summary of evidence | 19 | Summarize the main results (including an overview of concepts, themes, and types of evidence available), link to the review questions and objectives, and consider the relevance to key groups. | Discussion (pages 17–20) |
| Limitations | 20 | Discuss the limitations of the scoping review process. | Limitations (page 21) |
| Conclusions | 21 | Provide a general interpretation of the results with respect to the review questions and objectives, as well as potential implications and/or next steps. | Conclusion (pages 21–22) |
| Funding | 22 | Describe sources of funding for the included sources of evidence, as well as sources of funding for the scoping review. Describe the role of the funders of the scoping review. | Funding (page 22) |

Checklist based on Tricco, A. C., Lillie, E., Zarin, W., O’Brien, K. K., Colquhoun, H., Levac, D., Moher, D., Peters, M. D. J., Horsley, T., Weeks, L., Hempel, S., Akl, E. A., Chang, C., McGowan, J., Stewart, L., Hartling, L., Aldcroft, A., Wilson, M. G., Garritty, C., … Straus, S. E. (2018). PRISMA extension for scoping reviews (PRISMA-ScR): Checklist and explanation. Annals of Internal Medicine, 169(7), 467–473. https://doi.org/10.7326/M18-0850
