# Supplementary material for: Clear aligner interface properties, oral microbial ecology, and strategies for biofilm control and caries-risk mitigation: a scoping review
Source: Front Cell Infect Microbiol. 2026 Oct 23;16:1951083. doi: 10.3389/fcimb.2026.1951083 (PMC13741175; doi:10.3389/fcimb.2026.1951083)
Supplement: Supplementary file 2 [file Table2.docx]

**Supplementary Table S2. Database Search Strategies**

The database searches were conducted on 31 August 2026 and covered each database from inception to that date. No language or study-design restrictions were applied at the database level. The strategies combined a clear-aligner/appliance concept block with a microbial, biofilm, oral-ecology, caries-related, and preventive-intervention block. Controlled vocabulary and free-text terms were adapted to the syntax and searchable fields of each database. Line breaks below are for readability and do not alter the Boolean logic.

**1. PubMed/MEDLINE (PubMed)**

Search fields: Medical Subject Headings (MeSH) and Title/Abstract. Result count: 341 records. The complete high-sensitivity search strategy is reproduced below.

(

"clear aligner*"[Title/Abstract] OR "orthodontic aligner*"[Title/Abstract] OR "dental aligner*"[Title/Abstract] OR

"transparent aligner*"[Title/Abstract] OR "invisible aligner*"[Title/Abstract] OR "thermoplastic aligner*"[Title/Abstract] OR

"thermoformed aligner*"[Title/Abstract] OR "3D printed aligner*"[Title/Abstract] OR "3-D printed aligner*"[Title/Abstract] OR

"direct printed aligner*"[Title/Abstract] OR Invisalign[Title/Abstract] OR

(aligner*[Title/Abstract] AND (orthodont*[Title/Abstract] OR dental[Title/Abstract] OR tooth[Title/Abstract] OR teeth[Title/Abstract])) OR

aligner*[Title] OR "aligner material*"[Title/Abstract] OR "aligner sheet*"[Title/Abstract] OR "aligner tray*"[Title/Abstract] OR

"clear overlay appliance*"[Title/Abstract] OR "clear orthodontic appliance*"[Title/Abstract] OR

"transparent orthodontic appliance*"[Title/Abstract] OR "invisible orthodontic appliance*"[Title/Abstract] OR

"clear dental brace*"[Title/Abstract] OR "invisible dental brace*"[Title/Abstract] OR

"clear orthodontic device*"[Title/Abstract] OR "transparent orthodontic device*"[Title/Abstract] OR

(orthodont*[Title/Abstract] AND (bioplastic*[Title/Abstract] OR "dental plastic*"[Title/Abstract])) OR

"orthodontic attachment*"[Title] OR (orthodontic[Title] AND attachment*[Title]) OR

(COA[Title/Abstract] AND (orthodont*[Title/Abstract] OR dental[Title/Abstract]))

)

AND

(

"Biofilms"[Mesh] OR "Dental Plaque"[Mesh] OR "Microbiota"[Mesh] OR "Dental Caries"[Mesh] OR

"Tooth Demineralization"[Mesh] OR biofilm*[Title/Abstract] OR "bacterial adhesion"[Title/Abstract] OR

"microbial adhesion"[Title/Abstract] OR "microbial colonization"[Title/Abstract] OR

"bacterial colonization"[Title/Abstract] OR bacteri*[Title/Abstract] OR microbial[Title/Abstract] OR

microorganism*[Title/Abstract] OR microbiom*[Title/Abstract] OR microbiota[Title/Abstract] OR

metagenom*[Title/Abstract] OR metabolom*[Title/Abstract] OR transcriptom*[Title/Abstract] OR

mycobiom*[Title/Abstract] OR dysbios*[Title/Abstract] OR Streptococcus[Title/Abstract] OR

Lactobacill*[Title/Abstract] OR Candida[Title/Abstract] OR plaque[Title/Abstract] OR caries[Title/Abstract] OR

cariogenic[Title/Abstract] OR demineral*[Title/Abstract] OR remineral*[Title/Abstract] OR

"white spot lesion*"[Title/Abstract] OR "white-spot lesion*"[Title/Abstract] OR WSL[Title/Abstract] OR

decalcif*[Title/Abstract] OR antimicrobial*[Title/Abstract] OR antibacterial*[Title/Abstract] OR

antifungal*[Title/Abstract] OR anti-biofilm*[Title/Abstract] OR antibiofilm*[Title/Abstract] OR

cleanser*[Title/Abstract] OR disinfect*[Title/Abstract] OR decontamin*[Title/Abstract] OR

sanitiz*[Title/Abstract] OR "biofilm removal"[Title/Abstract] OR "plaque removal"[Title/Abstract] OR

fluoride[Title/Abstract] OR "fluoride release"[Title/Abstract] OR "ion release"[Title/Abstract] OR

"calcium phosphate"[Title/Abstract] OR "amorphous calcium phosphate"[Title/Abstract] OR

NACP[Title/Abstract] OR pH-responsive[Title/Abstract] OR "pH responsive"[Title/Abstract] OR

acid-responsive[Title/Abstract] OR "acid responsive"[Title/Abstract] OR

microfluidic*[Title/Abstract] OR "organ-on-chip"[Title/Abstract] OR "oral-on-a-chip"[Title/Abstract] OR

"dynamic biofilm"[Title/Abstract] OR microhardness[Title/Abstract]

)

**2. Embase (Embase.com)**

Result count: 307 records. Emtree terms were exploded where indicated (/exp) and combined with title, abstract, and author-keyword fields (:ti,ab,kw).

(

'clear aligner*':ti,ab,kw OR 'orthodontic aligner*':ti,ab,kw OR 'dental aligner*':ti,ab,kw OR

'transparent aligner*':ti,ab,kw OR 'invisible aligner*':ti,ab,kw OR 'thermoplastic aligner*':ti,ab,kw OR

'thermoformed aligner*':ti,ab,kw OR '3d printed aligner*':ti,ab,kw OR '3-d printed aligner*':ti,ab,kw OR

'direct printed aligner*':ti,ab,kw OR 'directly printed aligner*':ti,ab,kw OR invisalign:ti,ab,kw OR

(aligner*:ti,ab,kw AND (orthodont*:ti,ab,kw OR dental:ti,ab,kw OR tooth:ti,ab,kw OR teeth:ti,ab,kw)) OR

aligner*:ti OR 'aligner material*':ti,ab,kw OR 'aligner sheet*':ti,ab,kw OR 'aligner tray*':ti,ab,kw OR

'clear overlay appliance*':ti,ab,kw OR 'clear orthodontic appliance*':ti,ab,kw OR

'transparent orthodontic appliance*':ti,ab,kw OR 'invisible orthodontic appliance*':ti,ab,kw OR

'clear orthodontic device*':ti,ab,kw OR 'transparent orthodontic device*':ti,ab,kw OR

(orthodont*:ti,ab,kw AND (bioplastic*:ti,ab,kw OR 'dental plastic*':ti,ab,kw)) OR

'orthodontic attachment*':ti OR (orthodontic:ti AND attachment*:ti) OR

(coa:ti,ab,kw AND (orthodont*:ti,ab,kw OR dental:ti,ab,kw))

)

AND

(

'biofilm'/exp OR 'dental plaque'/exp OR 'microflora'/exp OR 'dental caries'/exp OR

'tooth demineralization'/exp OR biofilm*:ti,ab,kw OR 'bacterial adhesion':ti,ab,kw OR

'microbial adhesion':ti,ab,kw OR 'microbial colonization':ti,ab,kw OR 'bacterial colonization':ti,ab,kw OR

bacteri*:ti,ab,kw OR microbial:ti,ab,kw OR microorganism*:ti,ab,kw OR microbiom*:ti,ab,kw OR

microbiota:ti,ab,kw OR metagenom*:ti,ab,kw OR metabolom*:ti,ab,kw OR transcriptom*:ti,ab,kw OR

mycobiom*:ti,ab,kw OR dysbios*:ti,ab,kw OR streptococcus:ti,ab,kw OR lactobacill*:ti,ab,kw OR

candida:ti,ab,kw OR plaque:ti,ab,kw OR caries:ti,ab,kw OR carious:ti,ab,kw OR cariogen*:ti,ab,kw OR

deminerali*:ti,ab,kw OR reminerali*:ti,ab,kw OR 'white spot lesion*':ti,ab,kw OR decalcif*:ti,ab,kw OR

antimicrobial*:ti,ab,kw OR antibacterial*:ti,ab,kw OR antifungal*:ti,ab,kw OR antibiofilm*:ti,ab,kw OR

'anti biofilm*':ti,ab,kw OR cleanser*:ti,ab,kw OR disinfect*:ti,ab,kw OR decontamin*:ti,ab,kw OR

sanitiz*:ti,ab,kw OR 'biofilm removal':ti,ab,kw OR 'plaque removal':ti,ab,kw OR fluoride:ti,ab,kw OR

'ion release':ti,ab,kw OR 'calcium phosphate':ti,ab,kw OR nacp:ti,ab,kw OR

'ph responsive':ti,ab,kw OR 'acid responsive':ti,ab,kw OR microfluidic*:ti,ab,kw OR

'organ on chip':ti,ab,kw OR 'oral on a chip':ti,ab,kw OR 'dynamic biofilm':ti,ab,kw

)

**3. Web of Science Core Collection**

Result count: 333 records. Topic (TS) and title (TI) fields were searched; proximity operators were used where indicated.

(

TS=("clear aligner*" OR "orthodontic aligner*" OR "dental aligner*" OR "transparent aligner*" OR

"invisible aligner*" OR "thermoplastic aligner*" OR "thermoformed aligner*" OR "3D printed aligner*" OR

"3-D printed aligner*" OR "direct printed aligner*" OR "directly printed aligner*" OR Invisalign OR

"aligner material*" OR "aligner sheet*" OR "aligner tray*" OR "clear overlay appliance*" OR

"clear orthodontic appliance*" OR "transparent orthodontic appliance*" OR "invisible orthodontic appliance*" OR

"clear orthodontic device*" OR "transparent orthodontic device*" OR

(aligner* NEAR/5 (orthodont* OR dental OR tooth OR teeth)) OR

(orthodont* NEAR/5 (bioplastic* OR "dental plastic*")) OR (COA NEAR/3 (orthodont* OR dental)))

OR TI=(aligner* OR "orthodontic attachment*")

)

AND TS=(biofilm* OR "dental plaque" OR plaque OR "bacterial adhesion" OR "microbial adhesion" OR

"microbial colonization" OR "bacterial colonization" OR bacteri* OR microbial OR microorganism* OR

microbiom* OR microbiota OR metagenom* OR metabolom* OR transcriptom* OR mycobiom* OR dysbios* OR

Streptococcus OR Lactobacill* OR Candida OR caries OR carious OR cariogen* OR deminerali* OR

reminerali* OR "white spot lesion*" OR decalcif* OR antimicrobial* OR antibacterial* OR antifungal* OR

antibiofilm* OR "anti-biofilm*" OR cleanser* OR disinfect* OR decontamin* OR sanitiz* OR

"biofilm removal" OR "plaque removal" OR fluoride OR "ion release" OR "calcium phosphate" OR NACP OR

"pH responsive" OR "acid responsive" OR microfluidic* OR "organ-on-chip" OR "oral-on-a-chip" OR

"dynamic biofilm")

**Supplementary citation searching**

Backward citation searching was performed by screening the reference lists of included reports and relevant reviews identified during the search process. Forward citation searching was conducted using the Google Scholar "Cited by" function for included reports. Records identified through these supplementary methods were deduplicated against the database records and assessed using the same eligibility criteria.

**Duplicate removal and record handling**

All database exports were imported into Zotero. Duplicate records were identified using reference-manager matching and were subsequently checked manually before screening. Record counts at each stage of study selection are reported in the PRISMA-ScR flow diagram (Figure 1).

**Abbreviations**

COA, clear overlay appliance; MeSH, Medical Subject Headings; NACP, nano-amorphous calcium phosphate; TS, Topic; TI, Title; WSL, white spot lesion.
